# DNA-Binding Capabilities and Anticancer Activities of Ruthenium(II) Cymene Complexes with (Poly)cyclic Aromatic Diamine Ligands

**DOI:** 10.3390/molecules26010076

**Published:** 2020-12-26

**Authors:** Mona S. Alsaeedi, Bandar A. Babgi, Magda H. Abdellattif, Abdesslem Jedidi, Mark G. Humphrey, Mostafa A. Hussien

**Affiliations:** 1 Department of Chemistry, Faculty of Science, King Abdulaziz University, P.O. Box 80203, Jeddah 21589, Saudi Arabia; m.s.alsaeedi@outlook.com (M.S.A.); ajedidi@kau.edu.sa (A.J.); maabdulaal@kau.edu.sa (M.A.H.); 2 Department of Chemistry, College of Science, Taif University, Al-Haweiah, P.O. Box 11099, Taif 21944, Saudi Arabia; 3 Chemistry Department, Deanship of Scientific Research, College of Sciences, Taif University, Al-Haweiah, P.O. Box 11099, Taif 21944, Saudi Arabia; m.hasan@tu.edu.sa; 4 Research School of Chemistry, Australian National University, Canberra ACT 2601, Australia; mark.humphrey@anu.edu.au; 5 Department of Chemistry, Faculty of Science, Port Said University, Port Said 42521, Egypt

**Keywords:** ruthenium(II) arene, DNA-binding, anticancer properties

## Abstract

Ruthenium(II) arene complexes of the general formula [RuCl(η^6^-*p*-cymene)(diamine)]PF_6_ (diamine = 1,2-diaminobenzene (**1**), 2,3-diaminonaphthalene (**2**), 9,10-diaminophenanthrene (**3**), 2,3-diaminophenazine (**4**), and 1,2-diaminoanthraquinone (**5**) were synthesized. Chloro/aqua exchange was evaluated experimentally for complexes **1** and **2**. The exchange process was investigated theoretically for all complexes, revealing relatively fast exchange with no significant influence from the polycyclic aromatic diamines. The calf thymus DNA (CT-DNA) binding of the complexes increased dramatically upon extending the aromatic component of the diamines, as evaluated by changes in absorption spectra upon titration with different concentrations of CT-DNA. An intercalation binding mode was established for the complexes using the increase in the relative viscosity of the CT-DNA following addition of complexes **1** and **2**. Theoretical studies showed strong preference for replacement of water by guanine for all the complexes, and relatively strong Ru–N_guanine_ bonds. The plane of the aromatic systems can assume angles that support non-classical interactions with the DNA and covalent binding, leading to higher binding affinities. The ruthenium arenes illustrated in this study have promising anticancer activities, with the half maximal inhibitory concentration (IC_50_) values comparable to or better than cisplatin against three cell lines.

## 1. Introduction

Clinically-approved platinum-based anticancer drugs, which have been used successfully but have limitations [1,2,3,4,5], have inspired research interest in developing therapeutic agents containing other transition metals [6,7]. Ruthenium complexes have been considered good anticancer candidates, with two Ru(III) complexes having undergone clinical trials: *trans*-[Ru(dmso)(imidazole)Cl_4_]^+^ (Nami-A) [8] and *trans*-[Ru(indazole)_2_Cl_4_]^+^ (KP1019) [9]. These complexes are effective in treating some platinum-resistant tumors, and their action in vivo is thought to proceed through the reduction of Ru(III) to Ru(II) [10]. Half-sandwich ruthenium(II) arene complexes have been studied recently as an alternative class of potential anticancer drugs, due to the outstanding anticancer effects of [RuCl(en)(η^6^-biphenyl)]^+^, especially against platinum-resistant tumors [11]. Ruthenium(II) arenes consist of a hydrophobic arene ligand and a hydrophobic metal, with two or three co-ligands that can be used to introduce some specific features [12]. There have been numerous studies of η^6^-arene Ru(II) complexes with the general formula [Ru(η^6^-arene)(L)X]^n+^, where L is bidentate ligands with variant donor atoms such as O^O [13,14,15], N^O [16], N^S [17], N^N [18], N^P [19], and P^P [20]. It has been shown that modification of the bidentate ligand has a major impact on the anticancer effects of the organo-ruthenium complexes. Introducing chelating ligands may control the rate of the Cl/H_2_O ligand exchange reactions and govern the reactivity toward biomolecules (enzymes, proteins, DNA, etc.) [21,22].

The studies of the ruthenium(II) arene complexes containing ethylene diamine have shown that these complexes exhibit high in vitro and in vivo anticancer effects on human ovarian A2780 cell lines [23,24,25,26,27]. The N7 of guanine in DNA coordinates with the ruthenium, and intercalation of the extended arene and hydrogen bonding of the ethylene diamine NH_2_ groups with the DNA occur [23,24,25,26,27]. The different interactions of these complexes with duplex DNA compared to cisplatin cause different structural distortions in DNA than in cisplatin, which explains their effectiveness on platinum-resistant tumors [28]. The [RuCl(η^6^-*p*-cymene)(1,2-diaminobenzene) complex has been reported to possess promising cytotoxicity against A2780 ovarian cancer cells (IC_50_ = 11 μM), which is lost following oxidation that results in the formation of the corresponding phenylene diimine complex [29]. The loss of activity can be linked to the lower rate of hydrolysis and the less stable adducts with guanine (9-EtG) of the phenylene diimine complex, which weakens its DNA binding. Th activities of Ru arene complexes are correlated with their fast chlorido/aqua ligand exchange because the hydrolyzed products subsequently form adducts with DNA model compounds [29,30].

Motivated by these studies on diamine-ligated complexes, a set of ruthenium(II) cymene complexes with variant diamine ligands was synthesized, in which the diamines possessed extended polycyclic ring systems to improve their DNA-binding capability. The DNA binding and the anticancer properties of the complexes were examined, highlighting the relationships between structures and their properties.

## 2. Results and Discussion

### 2.1. Synthesis and Characterization

**Scheme 1:**
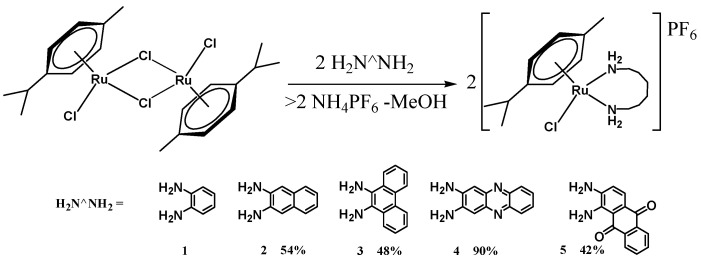
The synthesis of coordination compounds **1**–**5**.

A set of ruthenium(II) arene coordination compounds containing chloro and diamine ligands was targeted to assess their anticancer effects. The cationic ruthenium complexes were obtained through the reaction of the dimeric ruthenium precursor [Ru(*p*-cymene)(Cl)(µ-Cl)]_2_ with two molar equivalents of the diamine ligands in methanol in the presence of ammonium hexafluorophosphate (to exchange the resultant chloride counter anion with hexafluorophosphate) (Scheme 1). These complexes are insoluble in acetonitrile, chloroform, and diethyl ether, but soluble in DMSO. All these complexes were fully characterized by IR, NMR, elemental analysis, and mass spectrometry. The ^1^H-NMR spectra of the complexes showed resonances that agree with previously reported analogues [31,32]. All of the complexes exhibited a doublet at 0.76–0.86 ppm, which is assigned to the two methyl groups of the isopropyl substituent of the arene ligands, and singlet peaks at 1.83–1.90 ppm, which correspond to the three protons of the methyl in the *p*-cymene. In addition, septet peaks were seen at 2.39–2.57 ppm, which is correlated with the methine proton of the isopropyl group in the arene ligand, and two sets of doublets appeared between 5.22 and 5.92 ppm, which corresponds to the aromatic protons of the *p*-cymene. The resonances in the range of 6.75–8.25 ppm are assigned to the protons of the aromatic rings. The ^31^P-NMR spectra showed the typical septet around −140 ppm of the hexafluorophosphate counter-ion, with coupling constants between phosphorus and fluorine nuclei of ca. 700 Hz. The high-resolution mass spectra showed mass-to-charge peaks at 429.06615, 477.06644, 472.10660, and 509.05647 for the [M-PF_6_]^+^ ion for complexes **2**, **3**, **4**, and **5**, respectively.

### 2.2. Hydrolysis Process of the Complexes

**Figure 1:**
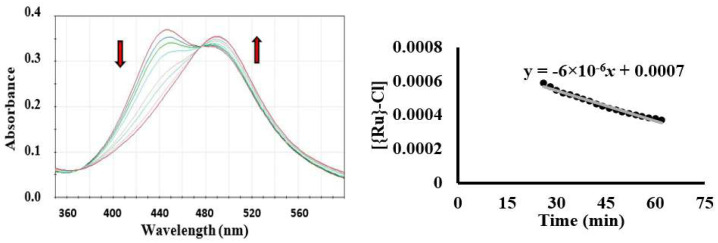
The changes in the absorption spectrum of complex **1,** showing chloro/aqua exchange upon dissolution in 10% water in DMSO.

**Figure 2:**
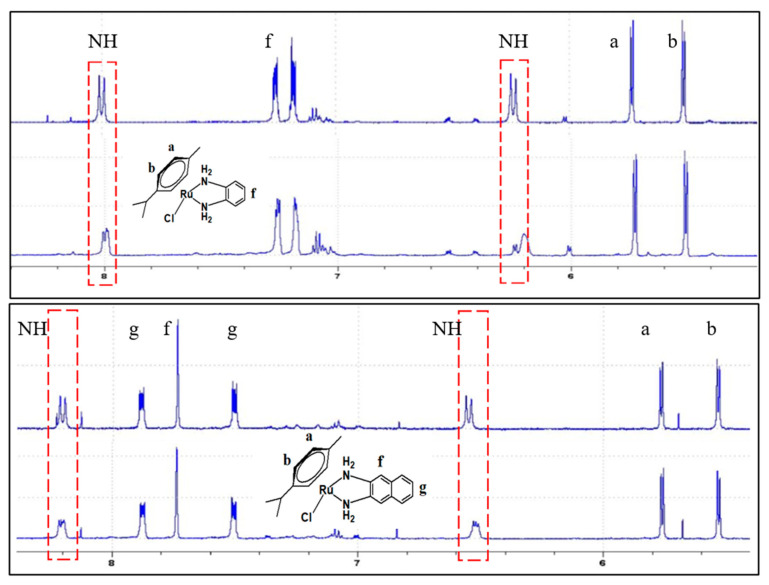
The changes in the ^1^H-NMR of complexes **1** (**top**) and **2** (**bottom**) 30 min after the addition of D_2_O to DMSO-*d*_6_ solutions of the complexes.

Complexes of the type [(η^6^-arene)Ru(H_2_N^NH_2_)Cl]^+^ displayed anticancer effects, including activity against cisplatin-resistant cancer cells [33,34]. In this type of complex, the arene plays an important role as the hydrophobic part, increasing the cytotoxicity and stabilizing the 2+ oxidation state. Such complexes undergo aqua/chloro exchange (similar to cisplatin), resulting in the aqua adducts (η^6^-arene)Ru(H_2_N^NH_2_)(H_2_O)]^2+^, which can form stable adducts with the *N7*-guanine units in the DNA [29]. In this study, the aqua/chloro ligand exchange processes of the ruthenium(II) arene complexes were monitored using UV–Vis spectroscopy. Complex **1** showed a relatively fast rate (6 × 10^−6^ min^−1^, Figure 1), with a slight decrease in the intensity of the peak at 448 nm and an increase in the peak at ca. 490 nm. The solution of the compound changed from yellow to brown over a period of ca. 90 minutes, which indicated that the ligand exchange occurred. The experimental determination of the rate for the other compounds was complicated by the strong overlaps between different absorption bands in the area of interest in the visible light region, although a clear indication of a color change from yellow to brown was noted for complex **2**. Changes were also noted in the ^1^H-NMR spectra of complexes **1** and **2** upon the addition of one drop of D_2_O to solutions of the compounds in DMSO-*d*_6_ (Figure 2). There were slight shifts in the N-H doublets located around 8.0 ppm and 6.2 ppm for complex **1** over a period of 30 min. Similarly, the N–H signals at ca. 8.2 ppm and 6.5 ppm for complex **2** exhibited shifts over the 30-min period. Due to this difficulty in calculating the rate of the aquation process experimentally, theoretical calculations were undertaken to evaluate whether the polycyclic aromatic backbones of the diamines had an impact on the rate of the chlorido/aqua exchange. The calculated ΔH for the aquation process of the five complexes suggested endothermic reactions that may be spontaneous at room temperature (as we observed experimentally for **1** and **2**) (Table 1). The effect of the polycyclic aromatic system appeared to be negligible when calculated using theoretical methods. Among the five complexes, complex **3** had the strongest and most stable Ru–OH_2_ bond (Table 1).

### 2.3. DNA-Binding Studies

**Figure 3:**
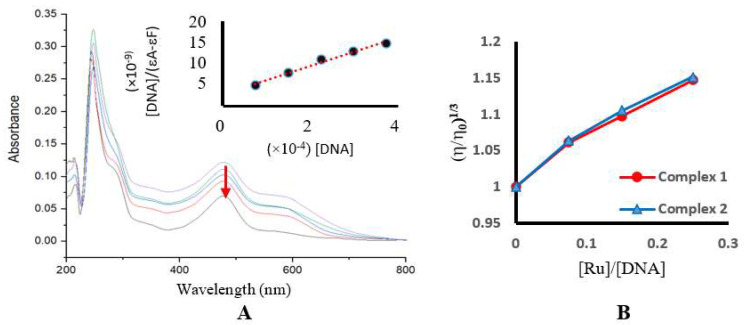
(**A**) The UV–vis spectroscopic changes at 482 nm of complex **3** upon increasing the concentration of CT-DNA. (**B**) The changes in the relative viscosity of DNA upon additions of different concentrations of complexes **1** and **2**.

**Figure 4:**
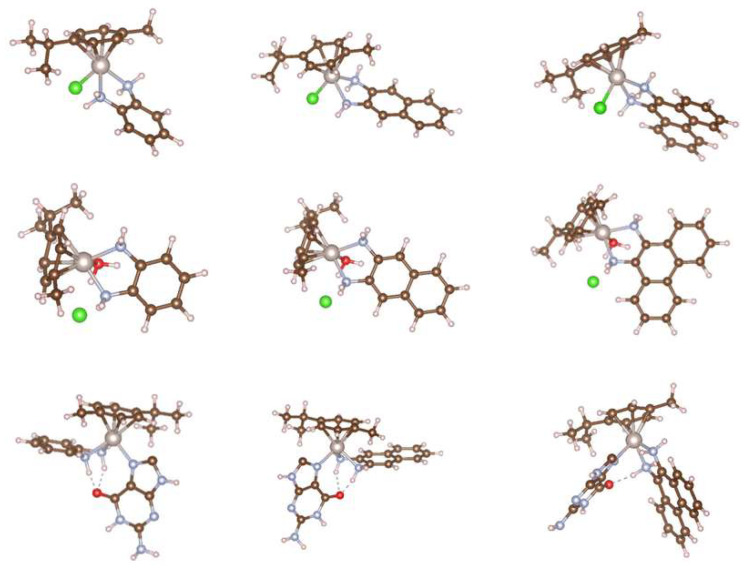
Optimized structures of complexes **1** (**left**), **2** (**middle**) and **3** (**right**) along with their aqua and guanine adducts, showing the changes in the plane angle of the aromatic system.

The motivation of our study was to increase the binding capability of complexes of the type [(η^6^-arene)Ru(H_2_N^NH_2_)Cl]^+^ by introducing the polycyclic aromatic units to the diamine ligand. For the calculation of intrinsic binding constant (K_b_), it was necessary to assume that only one type of interaction occurred between the CT-DNA and the compounds in the aqueous solution, resulting in the formation of one type of complex. The timeframe for the noncovalent interactions is fast, so the titration was performed to measure the noncovalent interactions of the molecules. The time required for the formation of the covalent bonding was quite large, so we ran the measurements within 5 min of each DNA addition to evaluate the changes in the π–π* or metal-to-ligand charge transfer (MLCT) bands. The binding affinities of the complexes were evaluated by following the changes in the spectra of the compounds upon increasing the concentration of the CT-DNA (Figure 3). DNA–compound interactions can be established via different modes, including covalent binding, minor groove binding, major groove binding, intercalation, and electrostatic interactions [35]. UV–vis absorption spectroscopy can assess the general interactions (overall binding affinity) between metal compounds and DNA [36]. All the compounds showed reductions in molar absorptivity of the π–π* and MLCT absorption bands (hypochromic shifts), indicating that binding had occurred. The calculated K_b_ values of the compounds are shown in Table 2. There was a twofold to threefold increase in the binding affinity upon introducing the highly delocalized aromatic systems, proceeding from **1** to complexes **2**, **3**, **4,** and **5**. In addition, changes of relative viscosity of CT-DNA were seen upon the addition of different concentrations of complexes 1 and 2 to evaluate their binding mode (Figure 3). The notable increases in the relative viscosity suggested that both compounds were intercalating. Since this class of complexes establishes its anticancer effects by coordinating to the *N7*-guanine units in the DNA [29,30,36,37], we investigated the bonding energies of complexes **1**–**5** toward guanine to assess the impact of the polyaromatic diamines. The calculated average bond enthalpies, which are a measure of the bond strengths, suggested that complex **3** had the most stable bond (–203.2 kcal/mol) followed by complexes **2** and **1** (Table 1). The heteroatoms in the aromatic systems of **4** and **5** were electron-withdrawing, which reduced the electron density around the metal due to weaker σ-donation from the amino ligands, resulting in weaker Ru–guanine bonds. Moreover, the position of the plane of the aromatic fragment of the diamine ligands relative to the ruthenium center (Figure 4) appeared to contribute to improvement in the binding affinity, since the plane of the aromatic systems and/or the cymene can establish π-stacking interactions with the DNA (see the position of the aromatic system relative to the guanine in Figure 3) [37]. These increases can possibly be attributed to the interactions of the extended π-electron systems with the DNA through stacking, intercalation, and/or minor groove binding [38] (Figure 3).

### 2.4. Anticancer Studies

The complexes were tested on three cell lines, and their efficiencies were compared to that of the clinically approved cisplatin (Table 3). Ovarian carcinoma is a widespread cancer for which cisplatin is normally prescribed following diagnosis (although this cell-type becomes platinum-resistant over time) [39]. The OVCAR-3 cell line is well-established and one of the most highly cited model systems for ovarian carcinoma. All complexes in our study exhibited IC_50_ values lower than that of cisplatin, with complexes **1** and **2** having the best cytotoxic effects. Skin cancer is another common cancer; the complexes were tested on a melanoma skin cell line (M-14) and found to have comparable or better anticancer activities to that of cisplatin. The anticancer activities also were evaluated against a non-small-cell lung cancer cell line (HOP-62), and the results suggested that complexes **2** and **3** have better cytotoxic effects than cisplatin.

## 3. Materials and Methods 

### 3.1. Materials

All the reactions were carried out in oven-dried glassware attached to a vacuum line and using standard Schlenk techniques in a nitrogen atmosphere. All the solvents and reagents were obtained from Sigma-Aldrich (St. Louis, MO, USA) and used without further purification. The experiments containing moisture-sensitive compounds were performed using solvents dried over A4 molecular sieves. All diamines were purchased from Alfa Aesar (Ward Hill, MA, USA) and used without further purification, and the ammonium hexafluorophosphate (NH_4_PF_6_) was purchased from Merck (Kenilworth, NJ, USA). The [Ru(*p*-cymene)(*o*-phenylenediamine)]PF_6_ was synthesized according to a standard procedure [40].

### 3.2. Methods and Instrumentation

The high-resolution electrospray ionization (ESI) mass spectra (positive ionization mode) were recorded at the Australian National University, using a Bruker Apex 4.7 FTICR-MS instrument (Billerica, MA, USA); all mass spectrometry peaks are reported as *m*/*z* (assignment). The elemental analyses were obtained at King Abdulaziz University. The infrared (IR) spectra were recorded using solid samples on a PerkinElmer Spectrum 100 instrument (Waltham, MA, USA); the peaks are reported in cm^−1^. The UV–Vis spectra were recorded in 1 cm quartz cells on a MultiSpec-1501 UV–Vis spectrophotometer (Kyoto, Japan) as chloroform solutions; the bands are reported in the form wavelength (nm) (extinction coefficient, 10^4^ M^−1^ cm^−1^). The UV–Vis emission spectra were recorded for nitrogen-purged chloroform solutions in 1 cm quartz cells using a PerkinElmer LS-55 fluorescence spectrometer; bands are reported in the form wavelength (nm). The ^1^H (850 MHz), ^31^P (344 MHz), and ^13^C (214 MHz) NMR spectra were obtained in CDCl_3_ solutions using a Bruker Avance 850 MHz spectrometer. The spectra were referenced to residual chloroform (7.26, ^1^H), CDCl_3_ (77.0, ^13^C), or external H_3_PO_4_ (0.0, ^31^P). 

### 3.3. Synthesis and Characterization

General synthetic protocol for [RuCl(*p*-cymene)(N^N)]PF_6_: We added 2.1 molar equivalents of the diamine ligand and 2.5 molar equivalents of NH_4_PF_6_ to a methanolic solution of [Ru(*p*-cymene)Cl_2_]_2_ in an N_2_ atmosphere. The reaction mixture was stirred for 18 h, and then cold diethyl ether (50 mL) was added. The resultant precipitate was collected by filtration to afford the product in reasonable yield.

*[RuCl(η^6^-p-cymene)(2,3-diaminonaphthalene)]PF_6_**(2)**:* [Ru(η^6^-*p*-cymene)Cl_2_]_2_ (105 mg, 0.17 mmol) was reacted with 2,3-diaminonaphthalene (56 mg, 0.35 mmol) and NH_4_PF_6_ (69 mg, 0.42 mmol) to obtain the product (**2**) as a yellow-green powder (105 mg, 54%). IR (KBr): 3232, 3306, 3400, 3552 cm^−1^ *v* (N–H stretching). ^1^H-NMR (850 MHz, DMSO-*d*_6_): (δ 7.60 (d, *J* = 6.00 Hz, 2H), 7.41 (s, 2H), 7.17 (d, *J* = 6.00 Hz, 2H), naphthalene), (5.48 (d, *J* = 6.00 Hz, 2H), 5.22 (d, *J* = 6.00 Hz, 2H), 2.57 (sept, *J* = 7.00 Hz, 1H), 1.90 (s, 3H), 0.86 (d, *J* = 7.00 Hz, 6H), *p*-cymene). ^13^C-NMR (214 MHz, DMSO-*d*_6_) δ 138.65, 131.13, 128.41, 127.35, 126.46, 124.50, 123.25, 121.25, 107.10, 102.48, 98.07, 86.40, 85.55, 82.31, 79.98, 30.09, 22.31, 21.53, 17.99. ^31^P-NMR (344 MHz, DMSO-*d*_6_): δ –144.21 (sept, *J* = 712, [PF_6_]^−^). HR ESI MS [C_20_H_24_N_2_ClRu]^+^: calcd: 429.06665, found 429.06615. Anal. Calcd for [C_20_H_23_N_2_Ru]^+^: calcd: 393.08991, found: 393.08969. Anal. Calcd for [C_20_H_24_N_2_Ru]^2+^: calcd: 197.04860, found: 197.04869. Anal. Calcd for C_20_H_24_ClF_6_N_2_PRu: C, 41.86; H, 4.22; N, 4.88%; found: C, 41.29; H, 3.81; N, 4.53%.

*[RuCl(η^6^-p-cymene)(9,10-diaminophenanthrene)]PF_6_***(3):** [Ru(η^6^-*p*-cymene)Cl_2_]_2_ (101 mg, 0.17 mmol) was reacted with 9,10-diaminophenanthrene (72 mg, 0.35 mmol) and NH_4_PF_6_ (67 mg, 0.41 mmol) to obtain the product (**3**) as a dark orange powder (97 mg, 48%). IR (KBr): 3236, 3415, 3552 cm^−1^ *v* (N–H stretching). ^1^H-NMR (850 MHz, DMSO-*d*_6_): (δ 8.29 (d, *J* = 8.00 Hz, 2H), 8.17 (d, *J* = 8.00 Hz, 2H), 7.48 (t, *J* = 8.00 Hz, 2H), 7.41 (t, *J* = 8.00 Hz, 2H), phenanthrene), (5.92 (d, *J* = 6.00 Hz, 2H), 5.71 (d, *J* = 6.00 Hz, 8H), 2.40 (sept, *J* = 7.00 Hz, 1H), 1.89 (s, 3H), 0.76 (d, *J* = 7.00 Hz, 6H), *p*-cymene). ^13^C-NMR (214 MHz, DMSO-*d*_6_) δ 166.49, 134.05, 131.72, 129.59, 126.75, 124.92, 124.48, 108.46, 105.64, 88.51, 87.95, 86.37, 30.97, 21.96, 21.50, 18.93. ^31^P-NMR (344 MHz, DMSO-*d*_6_) δ –144.21 (sept, *J* = 712, [PF_6_]^−^). HR ESI MS [C_24_H_24_N_2_ClRu]^+^: calcd: 477.06644, found 477.06665 Anal. Calcd for [C_24_H_24_N_2_Ru]^2+^: calcd: 221.04860, found: 221.04834. Anal. Calcd for C_24_H_26_ClF_6_N_2_PRu: C, 46.20; H, 4.20; N, 4.49%; found: C, 45.77; H, 4.02; N, 4.62%.

*[RuCl(η^6^-p-cymene)(2,3-diaminophenazine)]PF_6_***(4):** [Ru(η^6^-*p*-cymene)Cl_2_]_2_ (125 mg, 0.20 mmol) was reacted with 2,3-diaminophenazine (90 mg, 0.43 mmol) and NH_4_PF_6_ (83 mg, 0.51 mmol) to obtain the product (**4**) as a purple powder (231 mg, 90%). IR (KBr): 3229, 3415, 3552 cm^−1^ *v* (N–H stretching). ^1^H-NMR (850 MHz, DMSO-*d*_6_): (δ 6.76 (m, 2H), 6.72 (m, 2H), 5.84 (s, 2H), phenazine), (5.44 (d, *J* = 6.00 Hz, 2H), 5.22 (d, *J* = 6.00 Hz, 2H), 2.39 (sept, *J* = 7.00 Hz, 1H), 1.83 (s, 2H), 0.82 (d, *J* = 7.00 Hz, 6H), *p*-cymene). ^13^C-NMR (214 MHz, DMSO-*d*_6_) δ 167.23, 137.48, 125.49, 123.68, 115.39, 103.25, 101.27, 92.86, 86.37, 85.52, 85.50, 83.29, 30.80, 29.98, 22.16, 21.51, 18.71, 17.88. ^31^P-NMR (344 MHz, DMSO-*d*_6_) δ –144.21 (sept, *J* = 712, [PF_6_]^−^). HR ESI MS [RuC_22_H_24_N_4_Cl]^+^: calcd: 472.10660, found 472.10696. Anal. Calcd for C_22_H_24_ClF_6_N_4_PRu: C, 42.21; H, 3.86; N, 8.95%; found: C, 41.79; H, 3.55; N, 8.61%.

*[RuCl(η^6^-p-cymene)(1,2-diaminoanthraquinone)]PF_6_***(5):** [Ru(η^6^-*p*-cymene)Cl_2_]_2_ (108 mg, 0.17 mmol) was reacted with 1,2-diaminoanthraquinone (88 mg, 0.37 mmol) and NH_4_PF_6_ (72 mg, 0.44 mmol) to obtain the product (**5**) as a blue-green powder (98 mg, 42%). IR (KBr): 3239, 3415, 3552 cm^−1^ *v* (N–H stretching). ^1^H-NMR (850 MHz, DMSO-*d*_6_) δ 6.79 (d, *J* = 8.00 Hz, 2H), 6.75 (d, *J* = 8.00 Hz, 2H), 5.49 (d, *J* = 6.00 Hz, 1H), 5.45 (d, *J* = 6.00 Hz, 1H), 1.92 (s, 3H), 0.84 (d, *J* = 7.00 Hz, 6H). ^13^C-NMR (214 MHz, DMSO-*d*_6_) δ 133.55, 133.34, 128.92, 126.19, 33.08, 28.69, 24.09, 21.59, 20.67. ^31^P-NMR (344 MHz, DMSO-*d*_6_) δ –144.21 (sept, J = 712, [PF_6_]^−^). HR ESI MS [C_24_H_24_N_2_O_2_ClRu]^+^: calcd: 508.9806, found 509.05647. Anal. Calcd for C_24_H_24_ClF_6_N_2_O_2_PRu: C, 44.08; H, 3.70; N, 4.28%; found: C, 43.83; H, 3.96; N, 3.92%.

### 3.4. Hydrolyses

The aqua/chloro ligand exchanges of the ruthenium(II) arene complexes were monitored using UV–vis spectroscopy. Solutions of the complexes with concentrations ca. 500 µM were prepared by dissolving them in dimethyl sulfoxide containing 10% water. The absorbance was recorded at 2 min intervals at selected wavelengths over a period of ca. 60 min at 298 K. Plots of the change in absorbance with time were fitted to the appropriate equation for pseudo-first-order kinetics to provide the rate constants.

### 3.5. DNA Binding Studies

Absorption spectra of the complexes in the presence of calf thymus DNA were recorded, and the DNA absorption was subtracted. Six solutions were prepared by maintaining the concentration of ruthenium complexes around 50 μM and varying the ratio of [DNA] to [Ru-compound] from 0 to 8.0. To compare the quantitative affinity of the complexes bound to DNA, the intrinsic-binding constant K_b_ can be calculated from the following equation based on the titration process:

$$\frac{\left[\mathrm{DNA}\right]}{\left(\varepsilon A-\varepsilon F\right)}=\frac{\left[\mathrm{DNA}\right]}{\left(\varepsilon B-\varepsilon F\right)}+\frac{1}{\mathrm{Kb}\left(\varepsilon B-\varepsilon F\right)}$$

where εA, εF, and εB correspond to the extinction coefficient for the copper complex at the specific addition of DNA, before the addition of DNA, and at the fully bound mode, respectively. By plotting [DNA]/(εA−εF) versus [DNA] (supplementary data), K_b_ is obtainable by dividing the slope on the intercept [41]. The viscosity measurements were conducting using an Ostwald viscometer. Micro volumes (10 µL) of copper complexes were added to a solution of CT-DNA in buffer in which the [Ru]/[DNA] ratio was maintained in the range of 0.02 to 0.2. The solutions were allowed to stand for 24 hours at 25 °C in a water bath before measurements were taken. The flow times of the solutions were recorded and replicated at least four times. The relative viscosities (η/η_o_)^1/3^ were plotted against [Ru]/[DNA], where η_o_ and η represent the specific viscosity of the CT-DNA alone and that of the CT-DNA-Ru, respectively. The specific viscosity η and η_o_ were calculated using the formula [(t−t_b_)/t_b_], where t is the observed flow time and t_b_ is the buffer flow time [41,42].

### 3.6. Computational Details

We performed our calculations using the Gaussian09 suite (Gaussian Inc, Wallingford, CT, USA) [43] and monitored our input and output files using Gaussview Software (Gaussian Inc, Wallingford, CT, USA) [44]. The density functional theory (DFT) method was applied with the B3LYP functional [45], with a split-valence double-zeta basis set (6-31G) and five d-type Cartesian Gaussian polarization functions on each of the atoms of C, N, O, Cl, and H [46], and the SDD basis set for Ru [47]. No symmetry constraints were used in the geometry optimizations, and the final geometries were confirmed to be the minimum potential energy structures through frequency calculations. Weak interactions have been included in the energy evaluations using Grimme D3 corrections [48].

### 3.7. Anticancer Activity and Cytotoxicity

The cells were bought from the Egyptian Holding Company for Biological Products and Vaccines (VACSERA, Giza, Egypt), and then maintained in a tissue culture unit. The cells were grown in Roswell Park Memorial Institute (RPMI)-1640 medium supplemented with 10% heat inactivated fatal bovine serum (FBS), 50 units/mL of penicillin, and 50 mg/mL of streptomycin and maintained in a humidified atmosphere containing 5% CO_2_ [49,50]. The cells were maintained as a monolayer culture using serial subculturing. The cell culture reagents were obtained from Lonza (Basel, Switzerland). The anticancer activities of the rested compounds were evaluated on OVCAR-3 (ovarian), M-14 (melanoma), and HOP-62 (non-small cell lung cancer) (lung cancer). Cytotoxicity was determined using the sulforhodamine B (SRB) assay method as previously described by Skehan et al. [51]. Exponentially growing cells were collected using 0.25% Trypsin-EDTA and seeded in 96-well plates at 1000–2000 cells/well in RBMI-1640 supplemented medium. After 24 h, the cells were incubated for 72 h with various concentrations of the tested compounds. Following 72 h of incubation, the cells were fixed with 10% trichloroacetic acid for 1 h at 4 °C. Wells were stained for 10 min at room temperature with 0.4% sulforhodamine B (SRBC) dissolved in 1% acetic acid. The plates were air-dried for 24 h, and the dye was solubilized with Tris-HCl for 5 min on a shaker at 1600 rpm. The optical density (OD) of each well was measured spectrophotometrically at 564 nm with an ELISA microplate reader (ChroMate-4300, Palm city, FL, USA). The IC_50_ values were calculated according to the equation for a Boltzman sigmoidal concentration response curve using nonlinear regression fitting models (Graph Pad, Prism Version 9, GraphPad Software, San Diego, CA, USA).

## 4. Conclusions

In this study, we described the synthesis of a set of ruthenium(II) arene complexes with the general formula [RuCl(η^6^-*p*-cymene)(diamine)]PF_6_, in which the diamine ligands possess extended polycyclic systems. The chlorido/aqua exchanges were evaluated experimentally for complex **1** using UV–Vis absorption spectra. The ^1^H-NMR spectra were collected for **1** and **2** after the addition of deuterium oxide to show changes after 30 min. Theoretical calculations for all the complexes showed relatively fast chlorido/aqua exchange, with no major influence from the polycyclic aromatic units of the diamine ligands. The CT-DNA binding of the complexes improved dramatically upon an increase in the delocalization of the aromatic fragment of the diamines, and an intercalation binding mode was established due to the increase in the CT-DNA’s relative viscosity upon the additions of the Ru compounds. The theoretical calculations showed a strong preference for all the complexes to replace water molecules with guanine; the plane of the aromatic system can be altered to allow for non-classical interaction modes with the DNA. The five ruthenium arenes illustrated in this study exhibited anticancer effects, with IC_50_ values comparable to or better than those of cisplatin against three cell lines (OVCAR-3, M-14, and HOP-62).

## Figures and Tables

**Table 1:** The theoretical analysis and bond energies of various complexes.

L		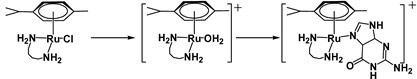						
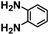	Ru (out of plane, Å)	0.529		−0.617			1.400	
	E_Ru-X_ (kcal/mole)	−11.7		−15.7			−190.7	
	ΔH (kcal/mole)		−1.6			−189.6		
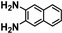	Ru (out of plane, Å)	0.876		−0.677			1.400	
	E_Ru-X_ (kcal/mole)	−11.9		−15.3			−191.3	
	E_diff_ (kcal/mole)		−3.8			−189.9		
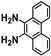	Ru (out of plane, Å)	0.288		−0.347			1.440	
	E_Ru-X_ (kcal/mole)	−13.5		−19.7			−203.2	
	E_diff_ (kcal/mole)		−1.4			−199.4		
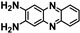	Ru (out of plane, Å)	0.722		1.350			1.400	
	E_Ru-X_ (kcal/mole)	−5.8		−15.7			−189.1	
	E_diff_ (kcal/mole)		−4.1			−188.9		
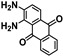	Ru (out of plane, Å)	0.787		1.220			1.340	
	E_Ru-X_ (kcal/mole)	−2.9		−10.6			−189.5	
	E_diff_ (kcal/mole)		−4.6			−188.4		

**Table 2:** The DNA binding affinities toward the CT-DNA measured in 5 mM Tris HCl buffer (pH = 7.4) and NaCl 50 mM at 295 K.

Compound	K_b_ (DNA Binding Constant) (by UV-Absorption)	Scaled K_b_ Value ^a^
**Ethidium Bromide**	6.67 × 10^4^	1.00
**1**	4.00 × 10^3^	0.06
**2**	7.50 × 10^3^	0.11
**3**	1.00 × 10^4^	0.15
**4**	1.14 × 10^4^	0.17
**5**	1.00 × 10^4^	0.15

^a^ The data are scaled by dividing the K_b_ value by that of ethidium bromide measured under the same conditions.

**Table 3:** Anticancer activities of selected compounds in DMSO solutions.

Complex	IC_50_ ± SD (μM)		
	OVCAR-3	M-14	HOP-62
**1**	4.31 ± 0.01	6.01 ± 0.02	4.12 ± 0.10
**2**	4.26 ± 0.01	6.09 ± 0.02	3.37 ± 0.10
**3**	5.19 ± 0.03	5.33 ± 0.02	3.51 ± 0.10
**4**	4.73 ± 0.02	6.03 ± 0.02	4.61 ± 0.16
**5**	4.89 ± 0.02	6.31 ± 0.02	3.89 ± 0.18
Cisplatin	5.89 ± 0.00	6.29 ± 0.05	3.91 ± 0.20

## Data Availability

The data presented in this study are available in supplementary material.

## Supplementary Materials

The following are available online, Figures S1–S37: Mass spectra, ^1^H NMR, ^31^P-NMR, ^13^C-NMR, and IR data of complexes **2**–**5**; Figure S38: The determination of the extinction coefficients of complexes **1**–**5**; Figures S39–S43: The titration of complexes **1**–**5** with CT-DNA to determine the binding affinity; Figures S44–S46: The cytotoxicity of complexes **1**–**5** against the cell lines; Figure S47: Inhabitation zones of the tested complexes at 1.25, 2.5, 5, 6.25, and 7.5 µM of the compounds.

## Sample Availability

Not available.

## Abbreviations

CT-DNA	Calf-thymus DNA
OVCAR-3	Ovarian carcinoma cancer cell line
M-14	Melanoma skin cancer cell line
HOP-62	Non-small-cell lung cancer cell line
