# Supplementary material for: DNA-Binding Capabilities and Anticancer Activities of Ruthenium(II) Cymene Complexes with (Poly)cyclic Aromatic Diamine Ligands
Source: Molecules. 2020 Dec 26;26(1):76. doi: 10.3390/molecules26010076 (PMC7795900; doi:10.3390/molecules26010076)
Supplement: Supplementary file 1 [file molecules-26-00076-s001.pdf]

Supporting Information

DNA-Binding Capabilities and Anticancer Activities of Ruthenium(II) Cymene Complexes with (Poly)cyclic Aromatic Diamine Ligands

Mona S. Alsaeedi [1,2], Bandar A. Babgi [1,*], Magda H. Abdellattif [3], Abdesslem Jedidi [1], Mark G. Humphrey [4] and Mostafa A. Hussien [1,5]

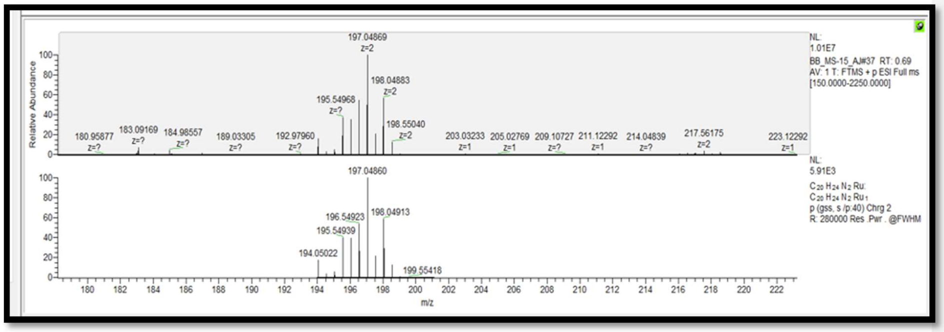

Figure S1: Mass spectrum of complex 2 -$[C_{20}H_{24}N_2ClRu]^+$

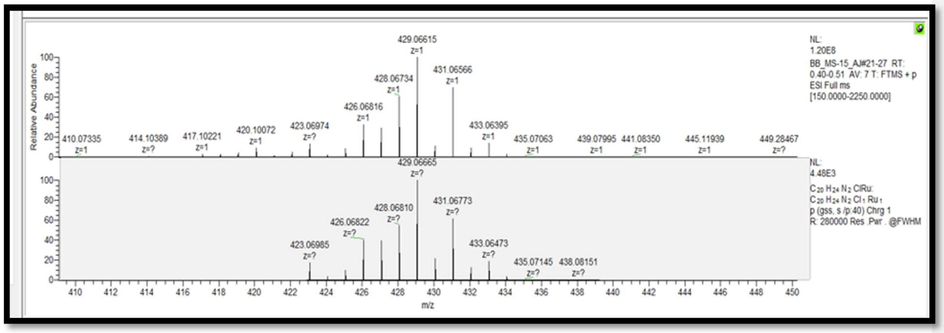

Figure S2: Magnified ESI+ Full scan Mass spectrum of the identified molecular ion at *m/z* 429.06.

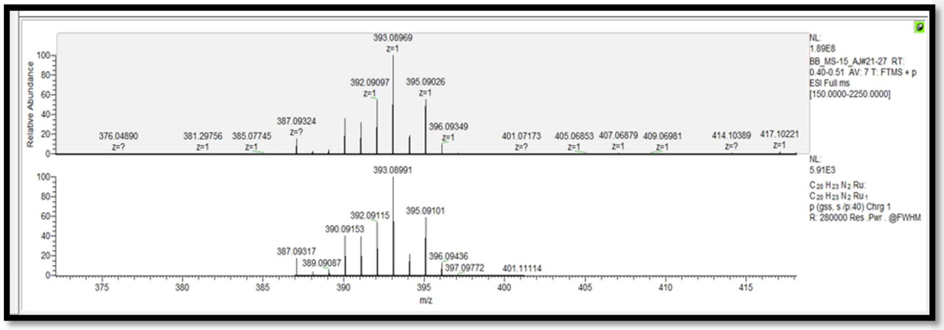

Figure S3: ESI+ Full scan Mass spectrum of $[C_{20}H_{23}N_2Ru]^+$

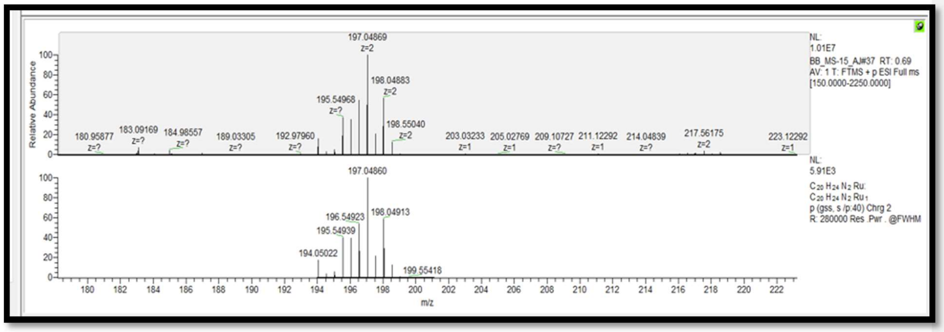

Figure S4: ESI+ Full scan Mass spectrum of $[C_{20}H_{24}N_2Ru]^{2+}$

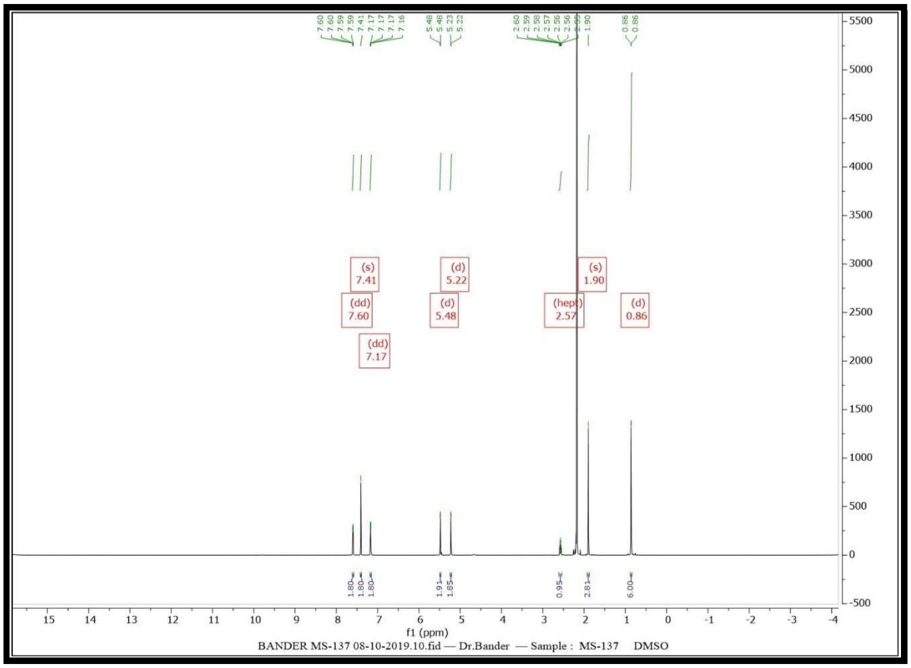

Figure S5: Full ^{1}H-NMR spectrum of $[(\eta^6$-*p*-cymene)Ru(2,3-diaminonaphthalene)]PF_6

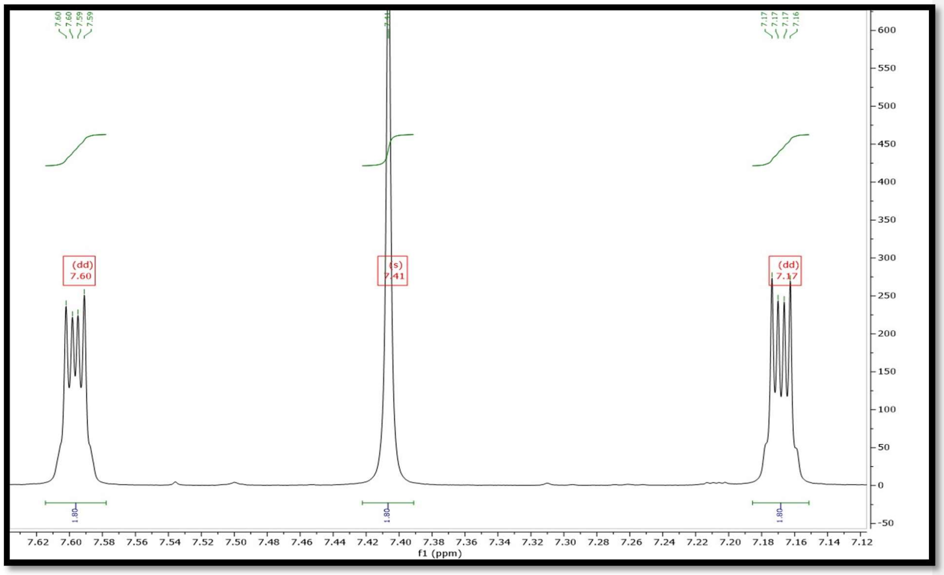

Figure S6: Magnified ^{1}H-NMR of [(η^6-*p*-cymene)Ru(2,3-diaminonaphthalene)]PF_6 [7.62-7.12ppm]

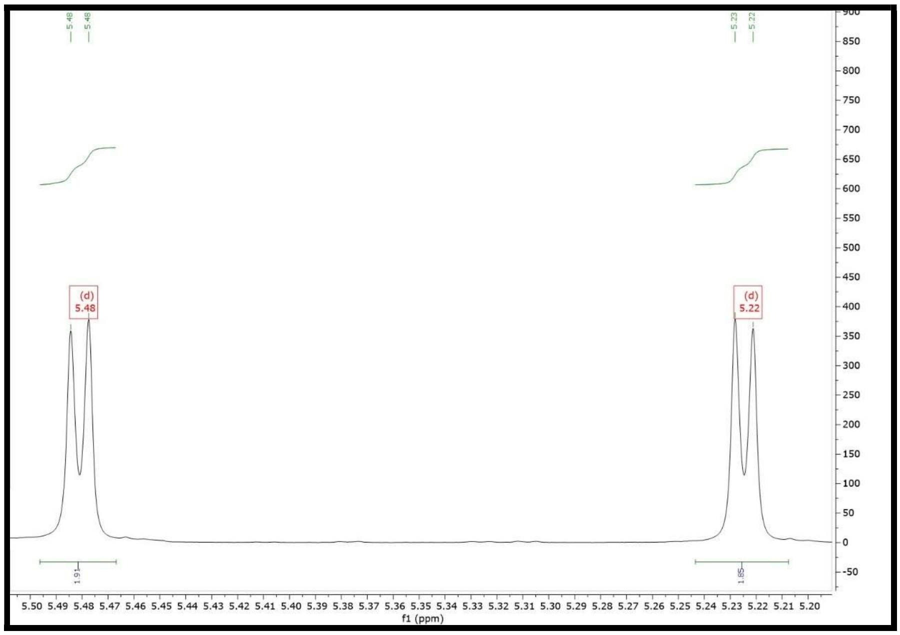

Figure S7: Magnified ^{1}H-NMR of [(η^6-*p*-cymene)Ru(2,3-diaminonaphthalene)]PF_6 [5.50-5.20ppm]

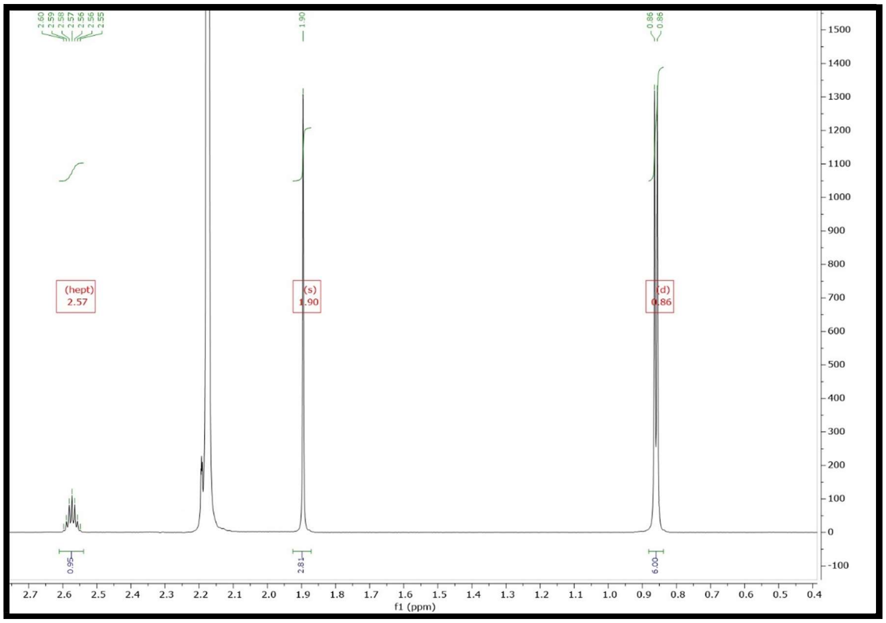

Figure S8: Magnified ^{1}H-NMR of [(η^6-*p*-cymene)Ru(2,3-diaminonaphthalene)]PF_6 [2.70-0.50ppm]

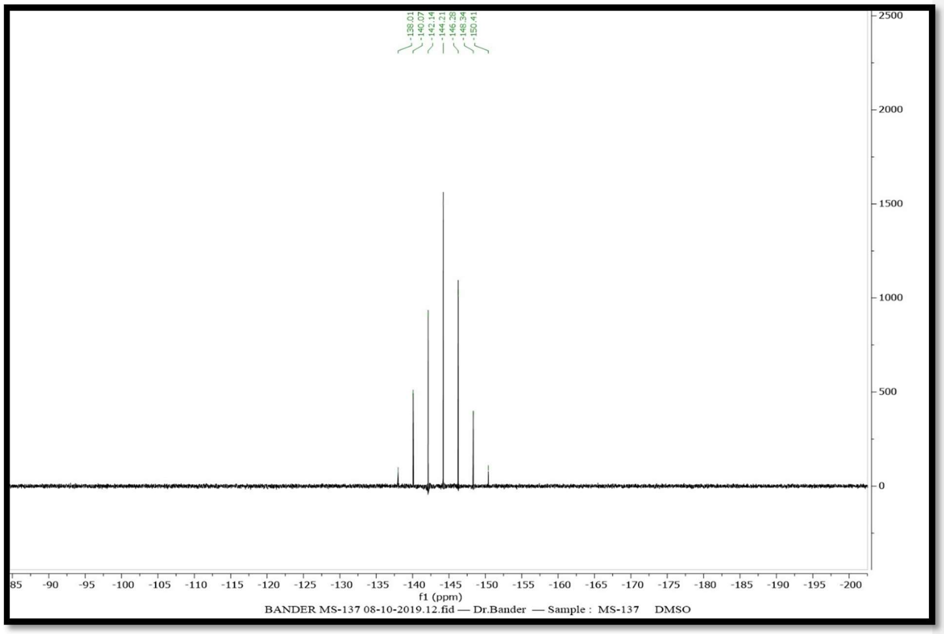

Figure S9: ^{31}P-NMR spectrum of [(η^6-*p*-cymene)Ru(2,3-diaminonaphthalene)]PF_6

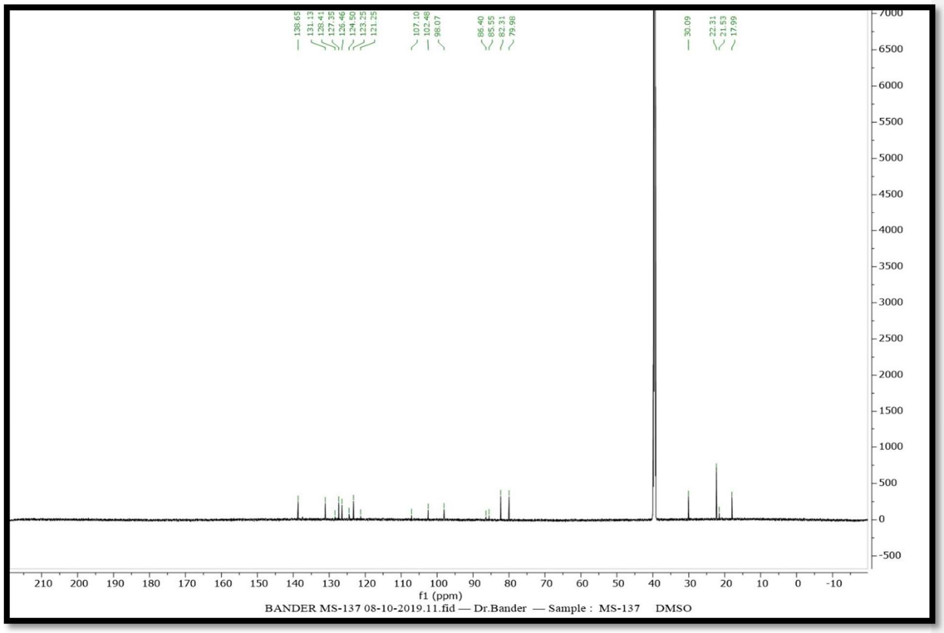

Figure S10: ^{13}C-NMR spectrum of [(η^6-*p*-cymene)Ru(2,3-diaminonaphthalene)]PF_6

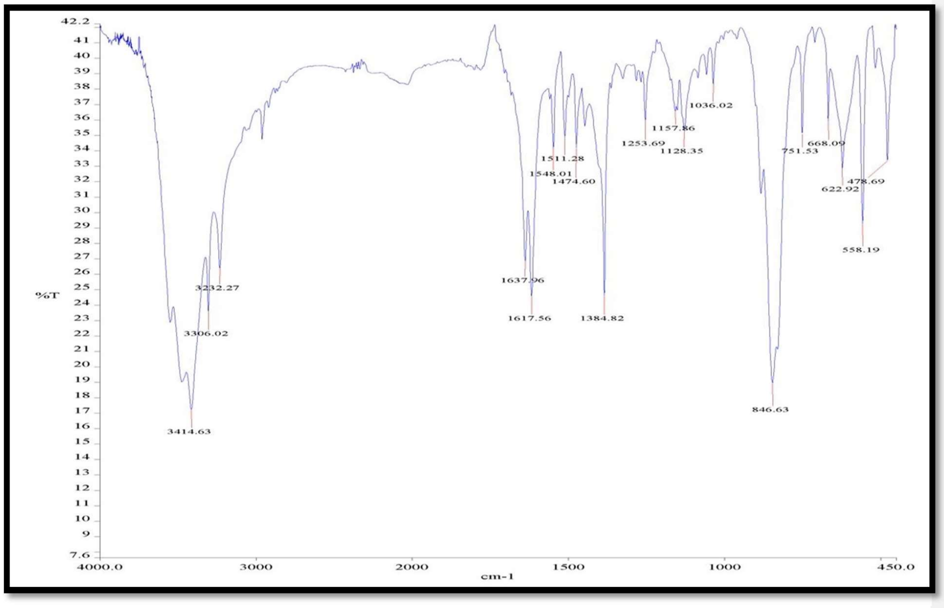

Figure S11: IR spectrum of [(η^6-*p*-cymene)Ru(2,3-diaminonaphthalene)]PF_6

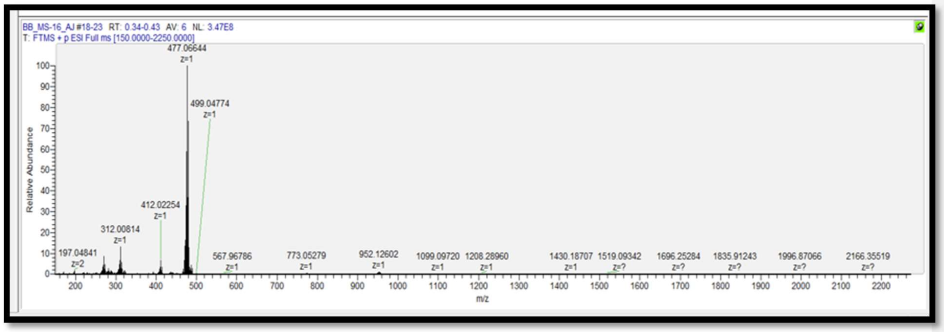

Figure S12: Mass spectrum of complex 3 - $[C_{24}H_{24}N_2ClRu]^+$

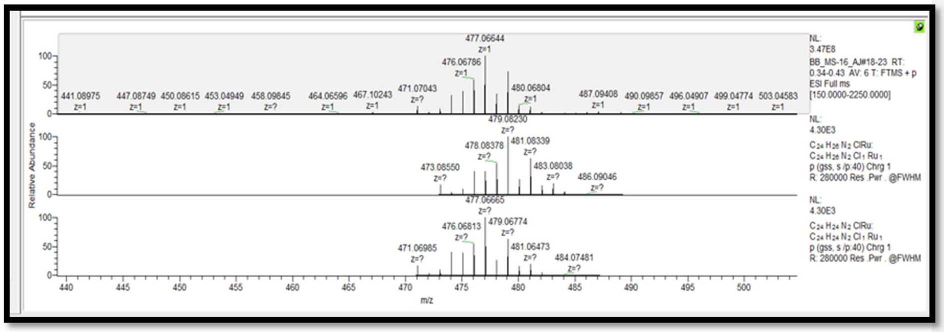

Figure S13: Magnified ESI+ Full scan Mass spectrum of the identified molecular ion at *m/z* 477.06.

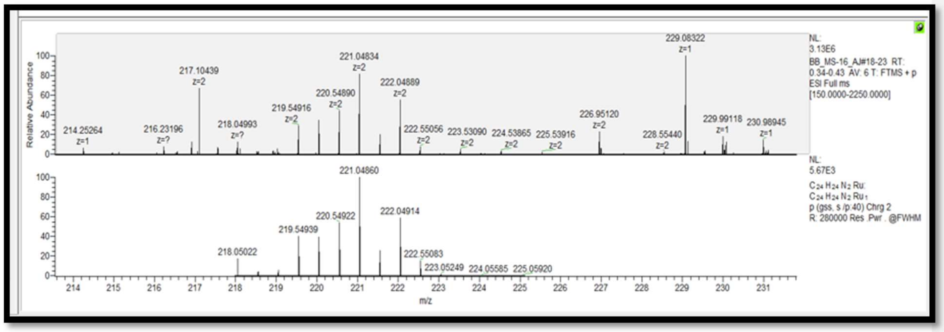

Figure S14: ESI+ Full scan Mass spectrum of $[C_{24}H_{24}N_2Ru]^{2+}$

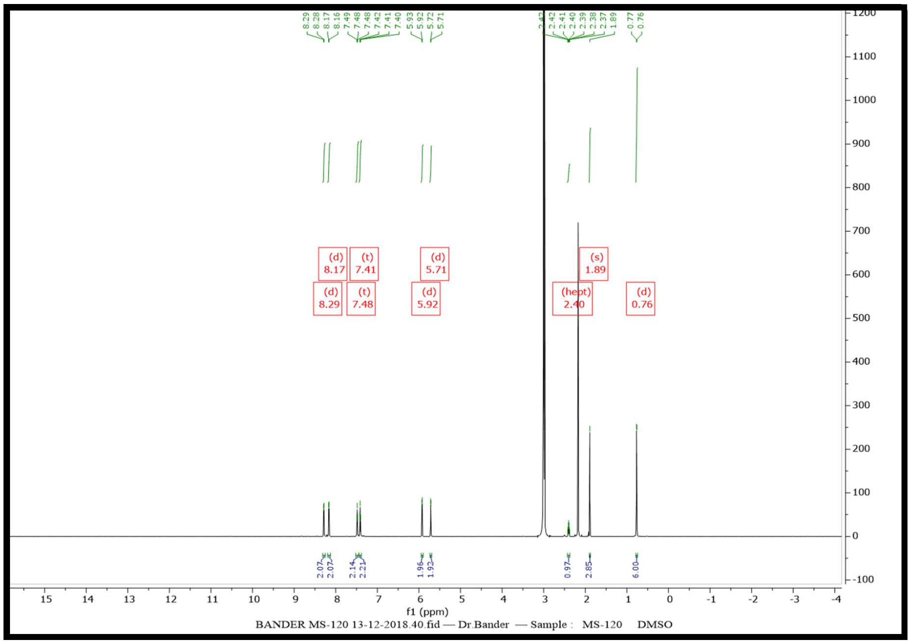

Figure S15: Full ^{1}H-NMR spectrum of [(η^6-*p*-cymene)Ru(9,10-diaminophenanthrene)]PF_6

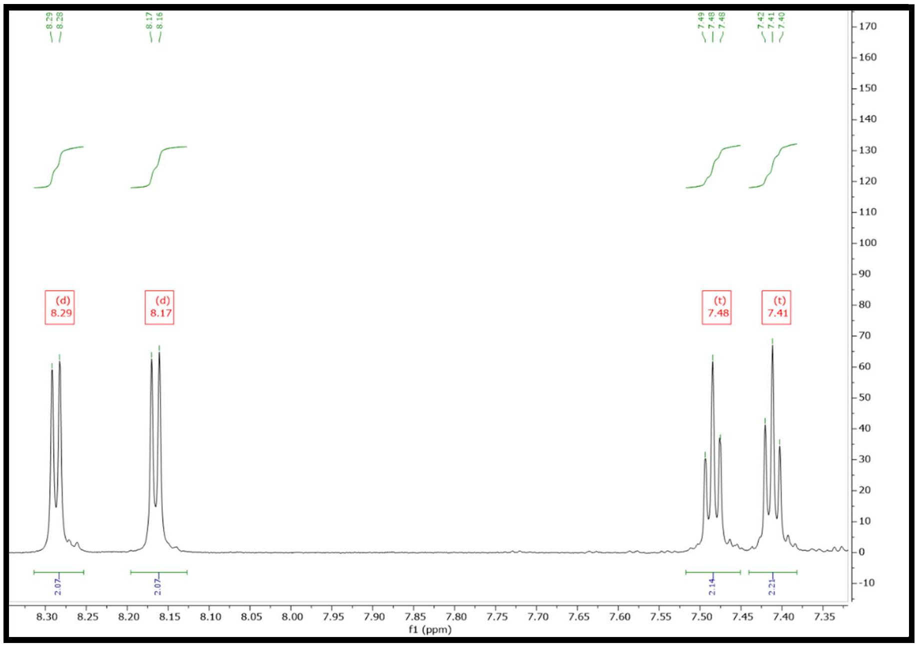

Figure S16: Magnified ^{1}H-NMR of[(η^6-*p*-cymene)Ru(9,10-diaminophenanthrene)]PF_6 [8.30-7.30ppm]

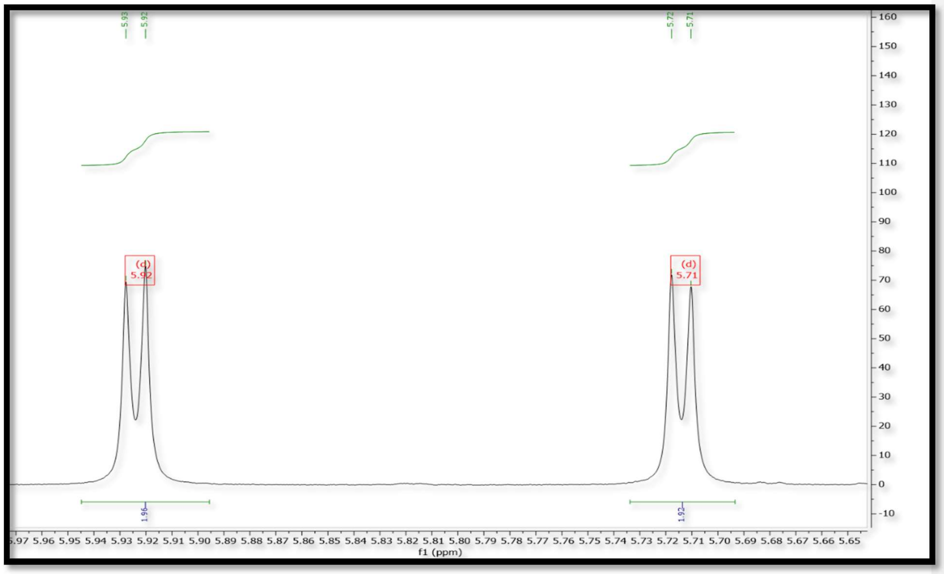

Figure S17: Magnified ^{1}H-NMR of[(η^6-*p*-cymene)Ru(9,10-diaminophenanthrene)]PF_6 [5.95-5.65ppm]

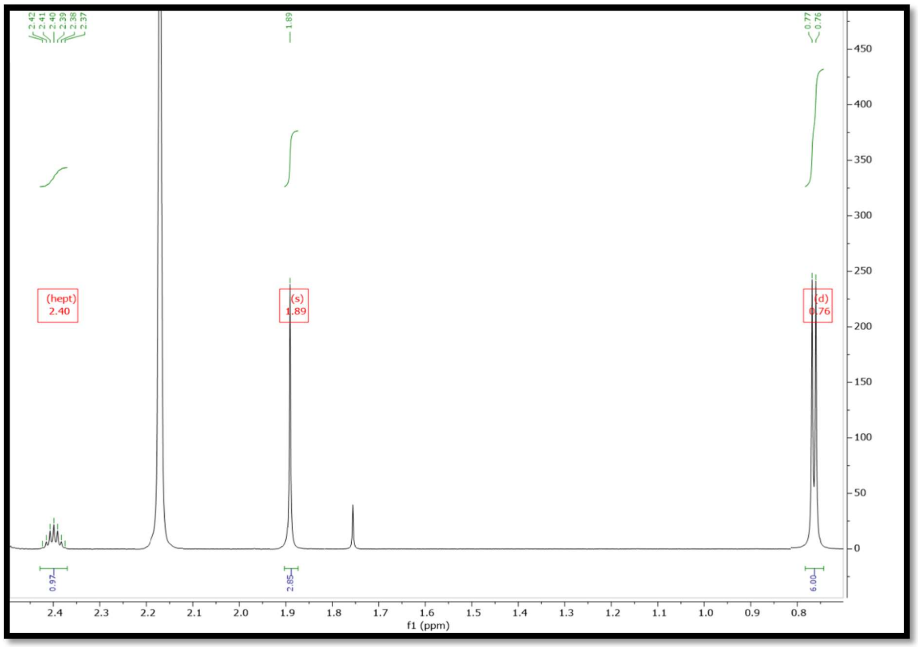

Figure S18: Magnified ^{1}H-NMR of[(η^6-*p*-cymene)Ru(9,10-diaminophenanthrene)]PF_6 [2.40-0.75ppm]

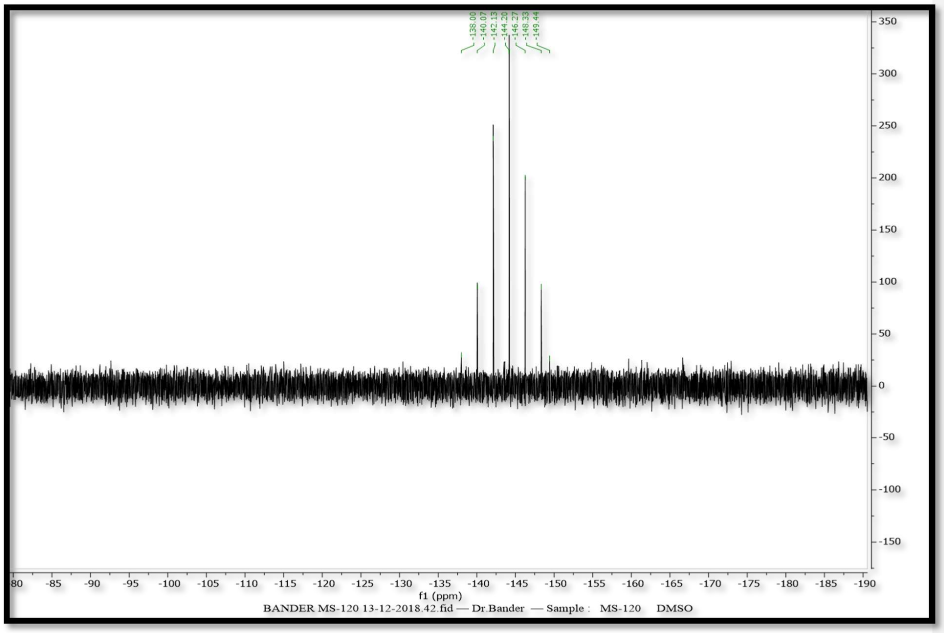

Figure S19: ^{31}P-NMR spectrum of [(η^6-*p*-cymene)Ru(9,10-diaminophenanthrene)]PF_6

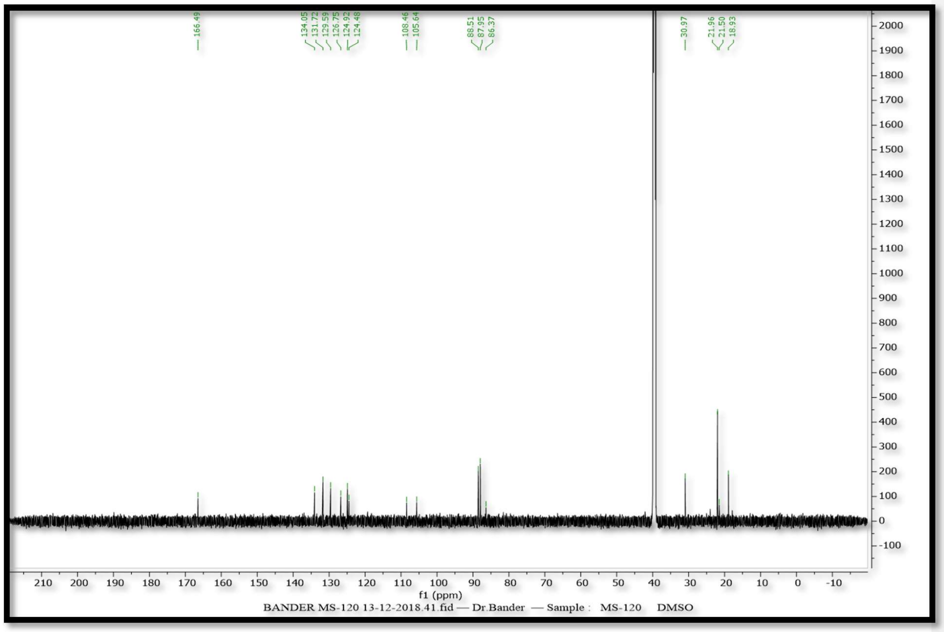

Figure S20: ^{13}C-NMR spectrum of [(η^6-*p*-cymene)Ru(9,10-diaminophenanthrene)]PF_6

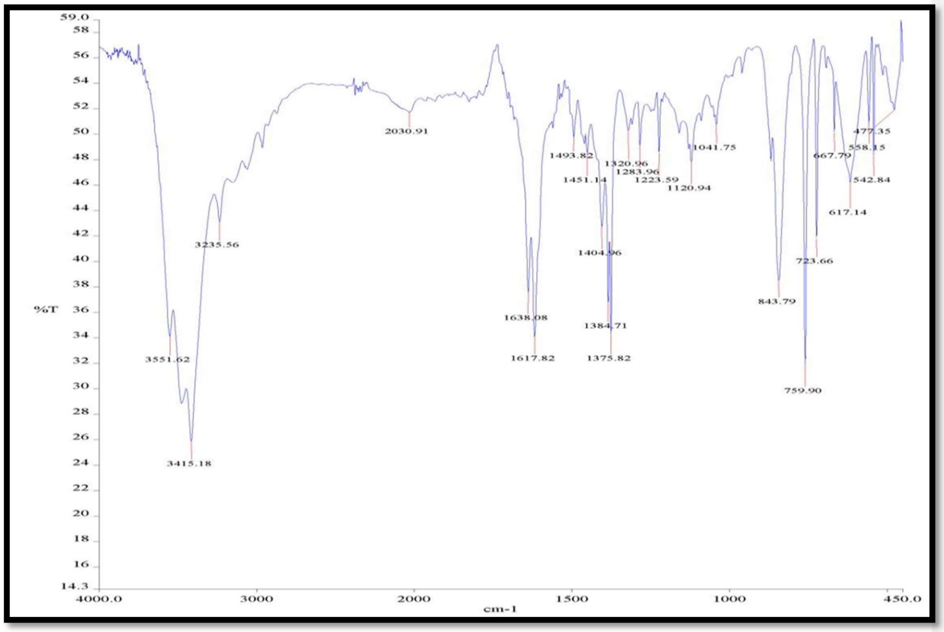

Figure S21: IR spectrum of [(η^6-*p*-cymene)Ru(9,10-diaminophenanthrene)]PF_6

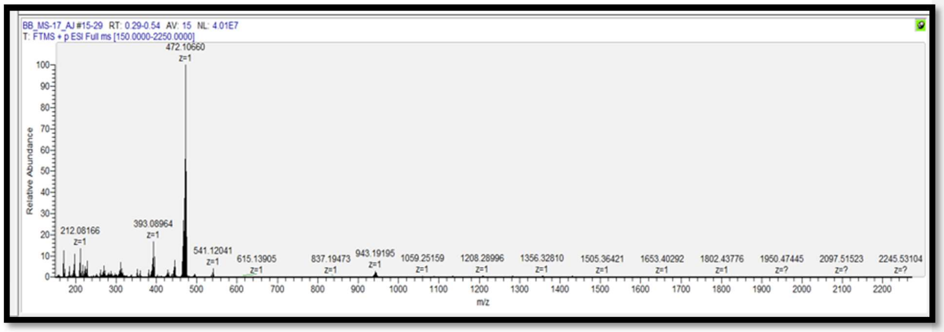

Figure S22: ESI+ Full scan Mass spectrum of complex 4 - $[RuC_{22}H_{24}N_4Cl]^+$

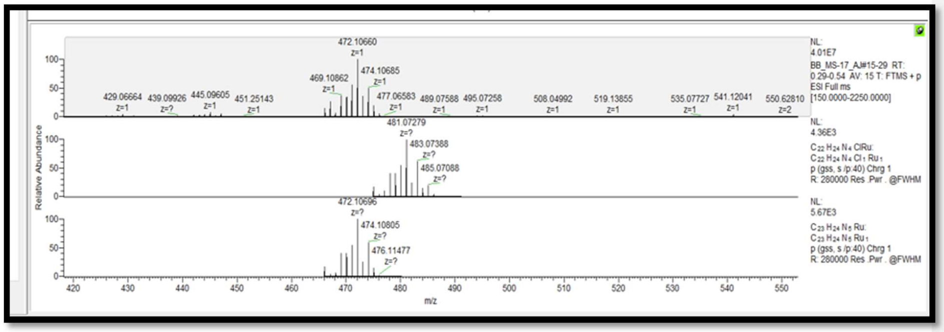

Figure S23: Magnified ESI+ Full scan Mass spectrum of the identified molecular ion at *m*/*z* 472.10.

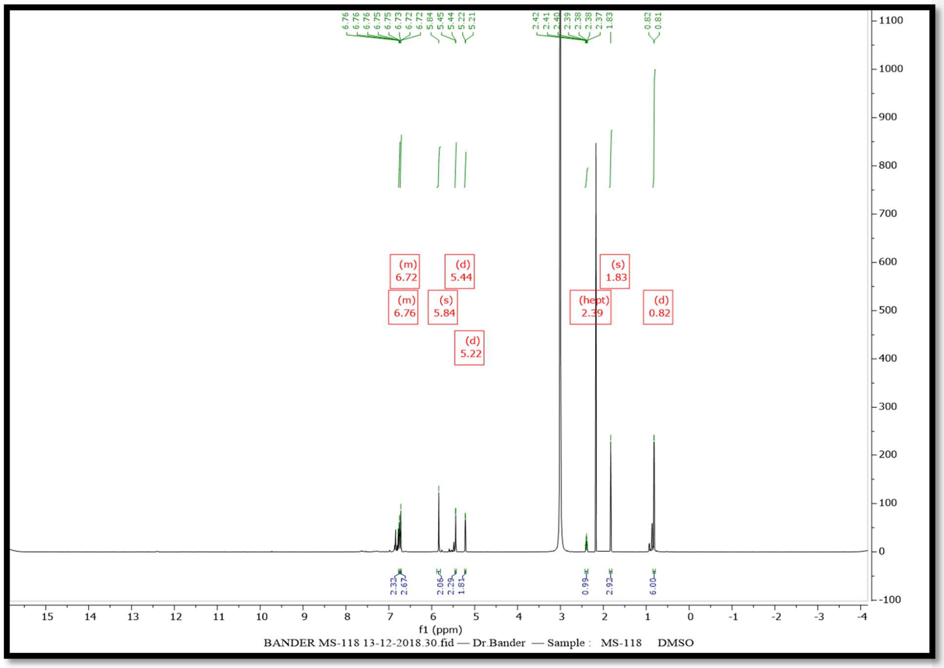

Figure S24: Full ^{1}H-NMR spectrum of [(η^{6}-*p*-cymene)Ru(2,3-diaminophenazine)]PF_6

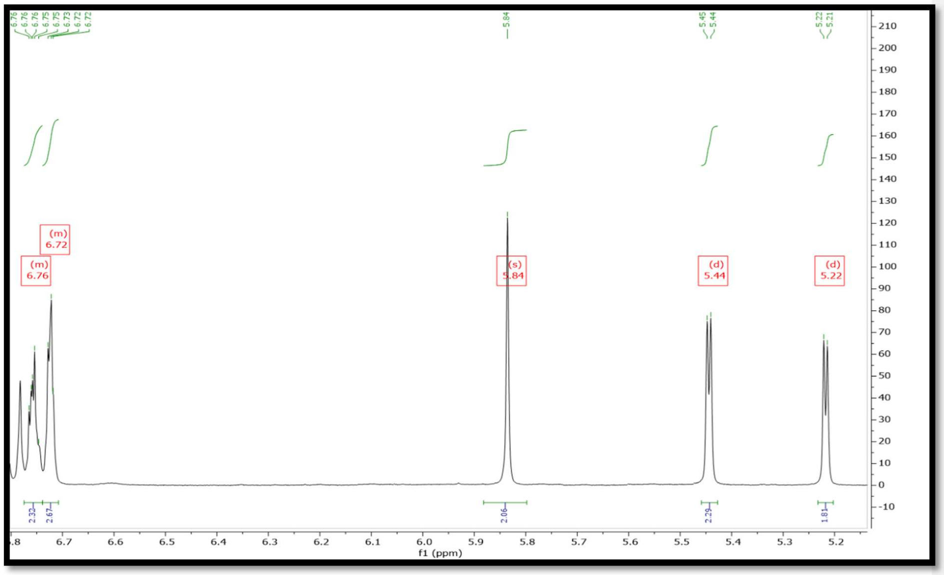

Figure S25: Magnified ^{1}H-NMR of [(η^6-*p*-cymene)Ru(2,3-diaminophenazine)]PF_6 [6.80-5.20ppm]

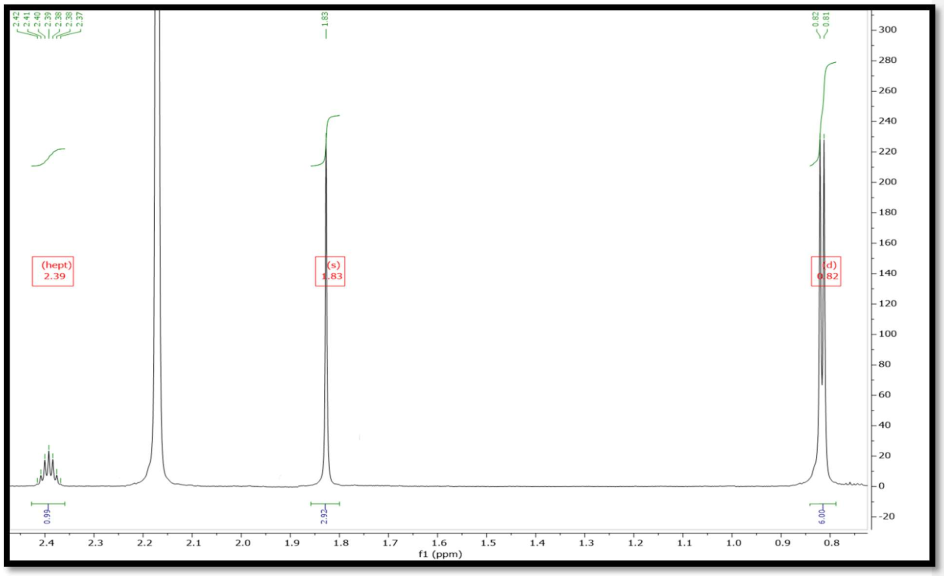

Figure S26: Magnified ^{1}H-NMR of [(η^6-*p*-cymene)Ru(2,3-diaminophenazine)]PF_6 [2.40-0.80ppm]

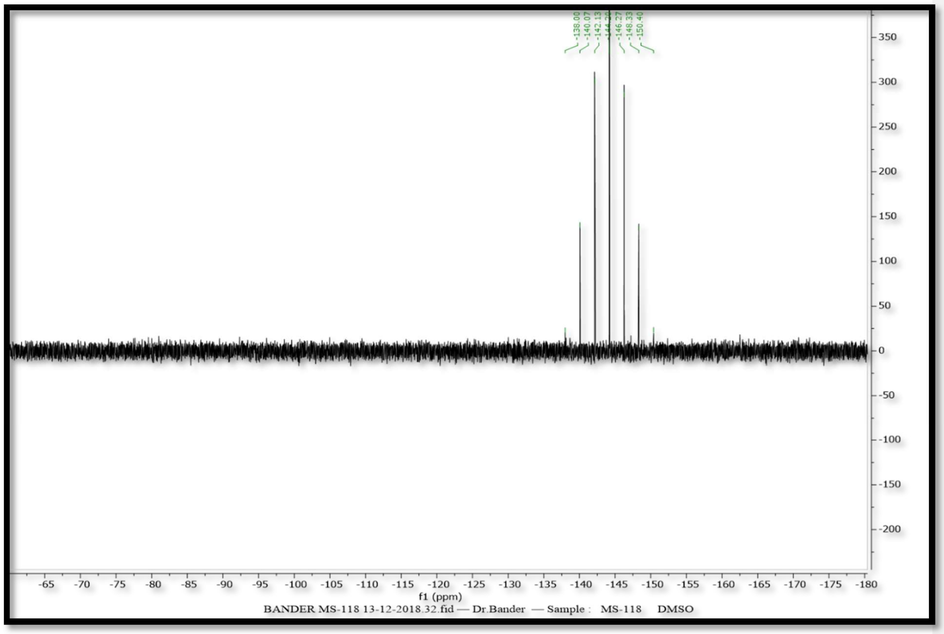

Figure S27: ^{31}P-NMR spectrum of [(η^6-*p*-cymene)Ru(2,3-diaminophenazine)]PF_6

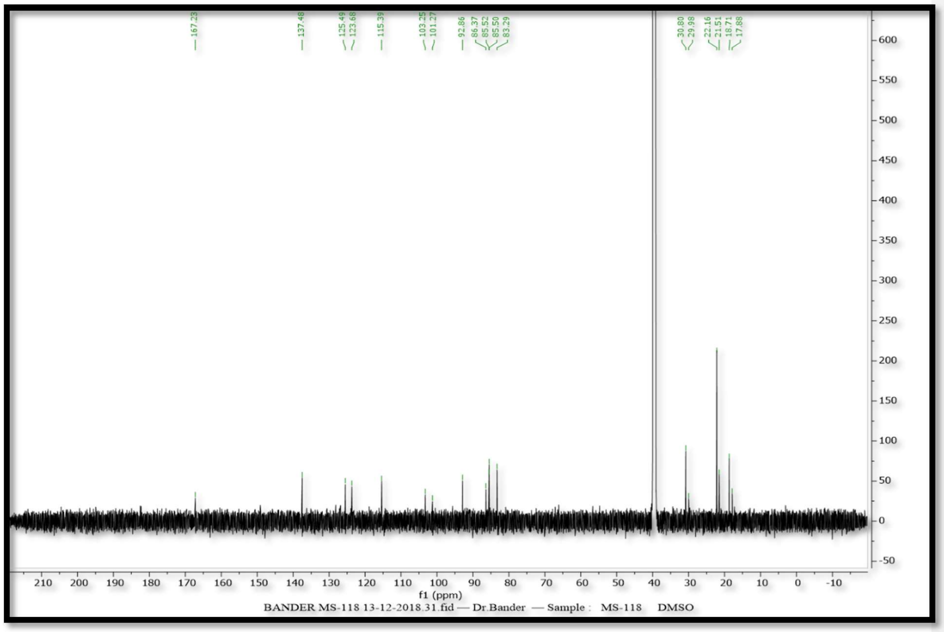

Figure S28: ^{13}C-NMR spectrum of [(η^6-*p*-cymene)Ru(2,3-diaminophenazine)]PF_6

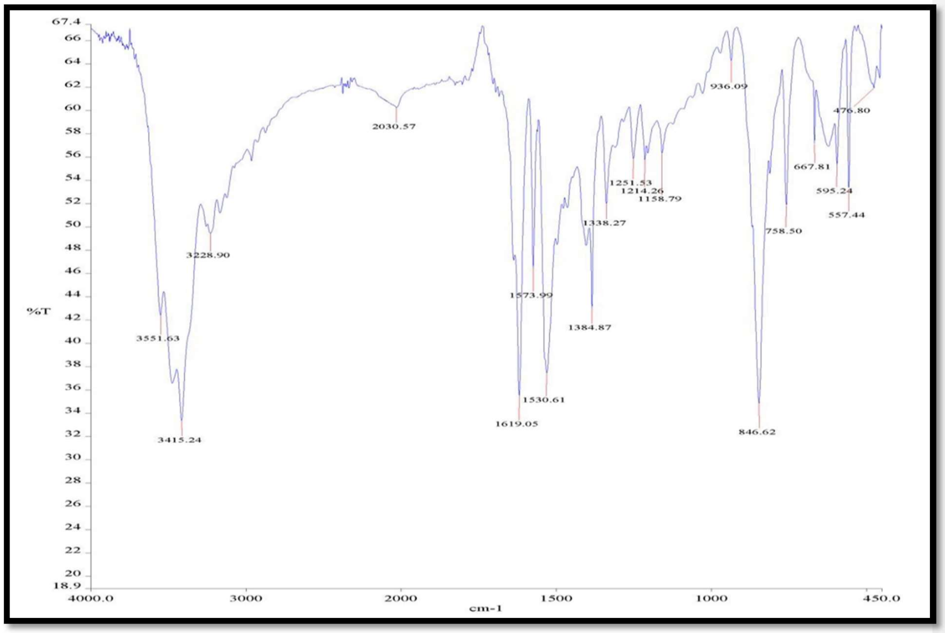

Figure S29: IR spectrum of [(η⁶-*p*-cymene)Ru(2,3-diaminophenazine)]PF_6

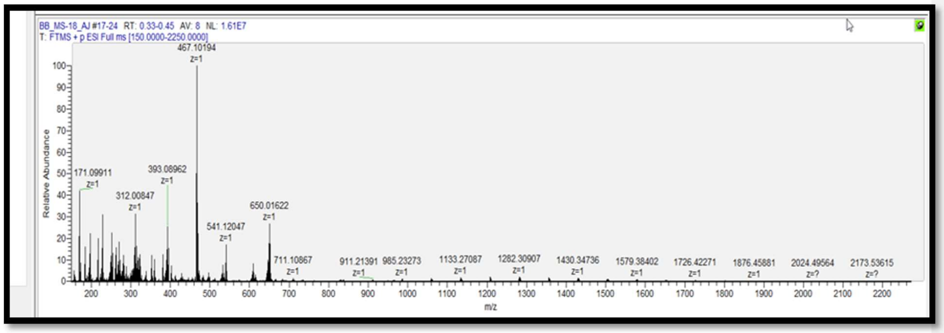

Figure S30: ESI+ Full scan Mass spectrum of complex 5 -$[C_{24}H_{24}N_2O_2ClRu]^+$

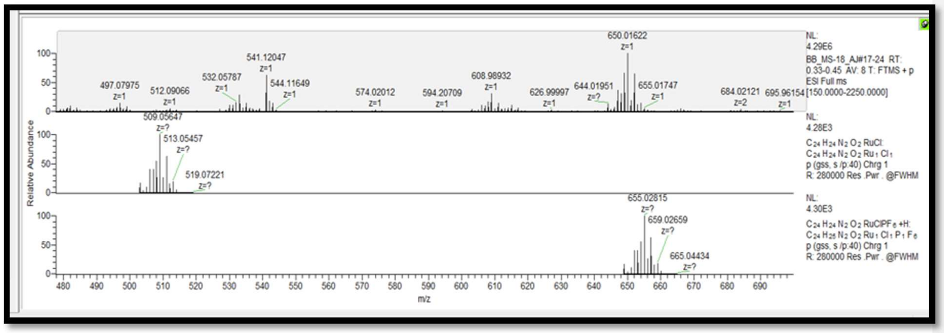

Figure S31: Mass spectrum of $[C_{24}H_{24}N_2O_2ClRu]^+$ and $[C_{24}H_{25}N_2O_2ClRuPF_6]^+$

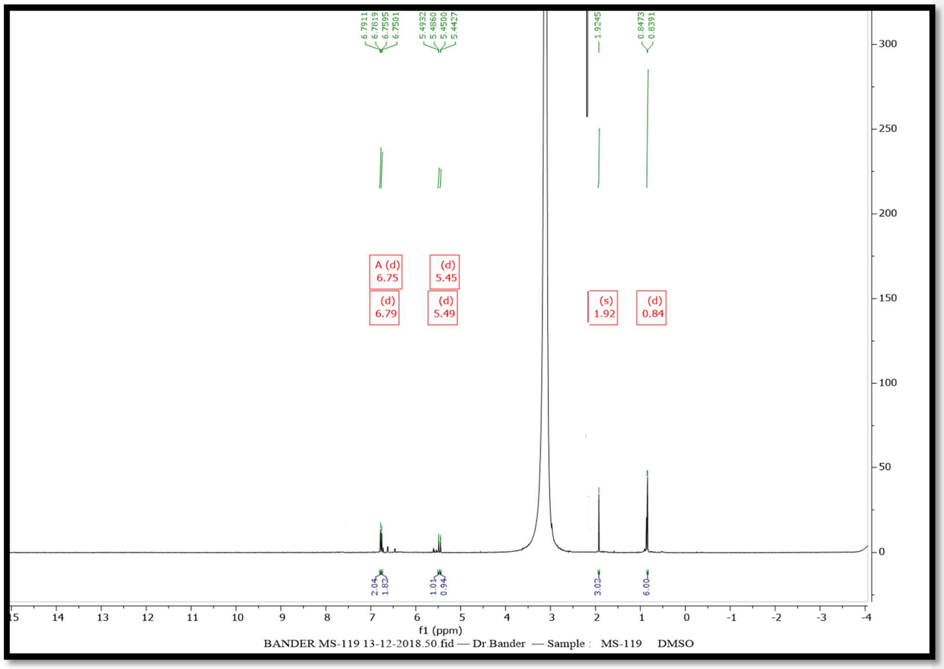

Figure S32: Full ^1H-NMR spectrum of [(η^6-*p*-cymene)Ru(1,2-diaminoanthraquinone)]PF_6

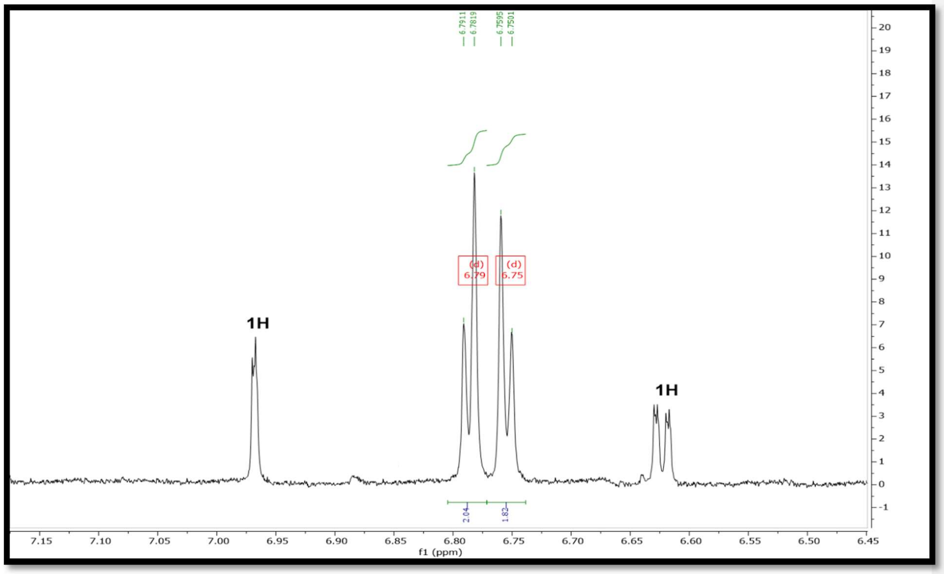

Figure S33: Magnified ^{1}H-NMR of [(η^{6}-*p*-cymene)Ru(1,2-diaminoanthraquinone)]PF_6 [7.15-6.45ppm]

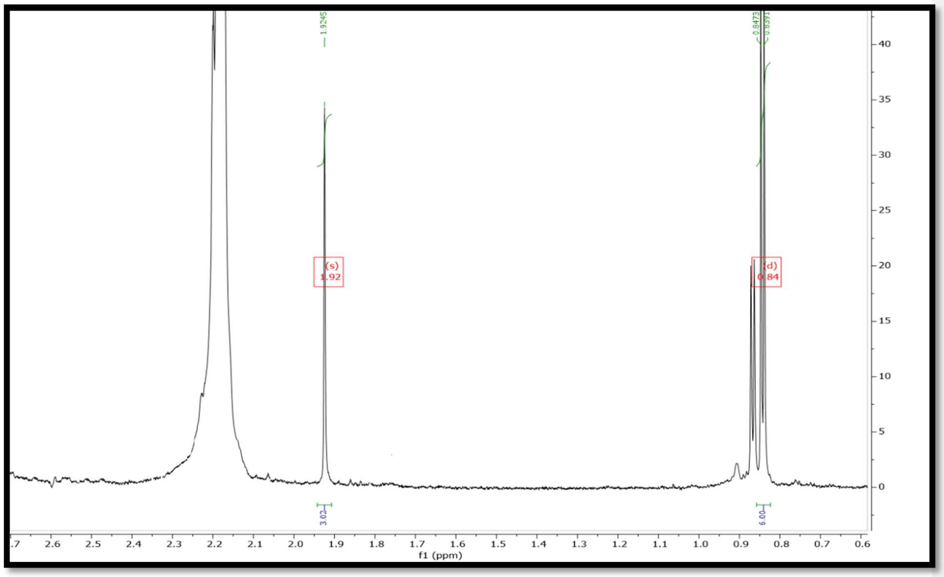

Figure S34: Magnified ^{1}H-NMR of [(η^{6}-*p*-cymene)Ru(1,2-diaminoanthraquinone)]PF_6 [2.50-0.70ppm]

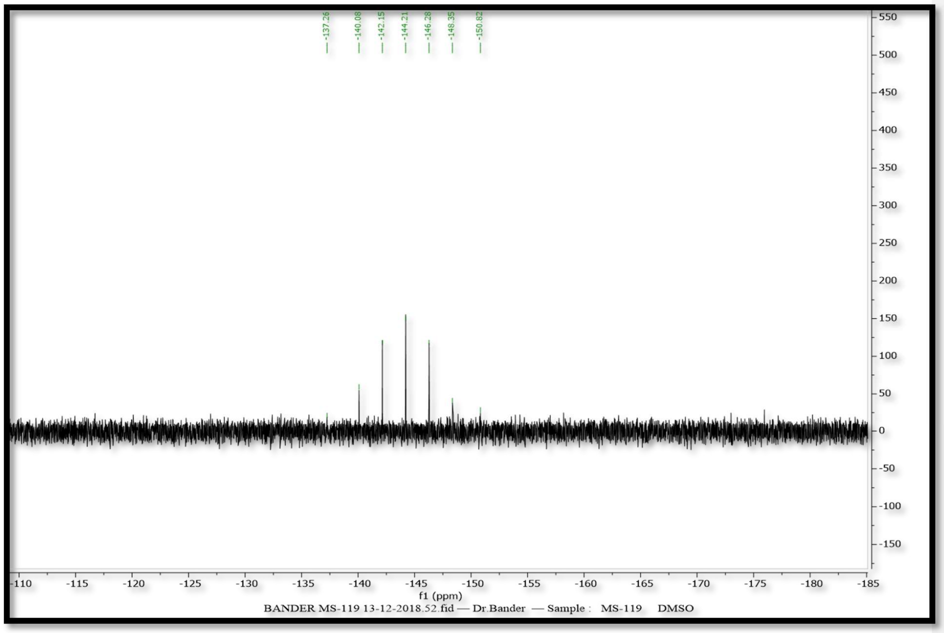

Figure S35: ^{31}P-NMR spectrum of [(η^6-*p*-cymene)Ru(1,2-diaminoanthraquinone)]PF_6

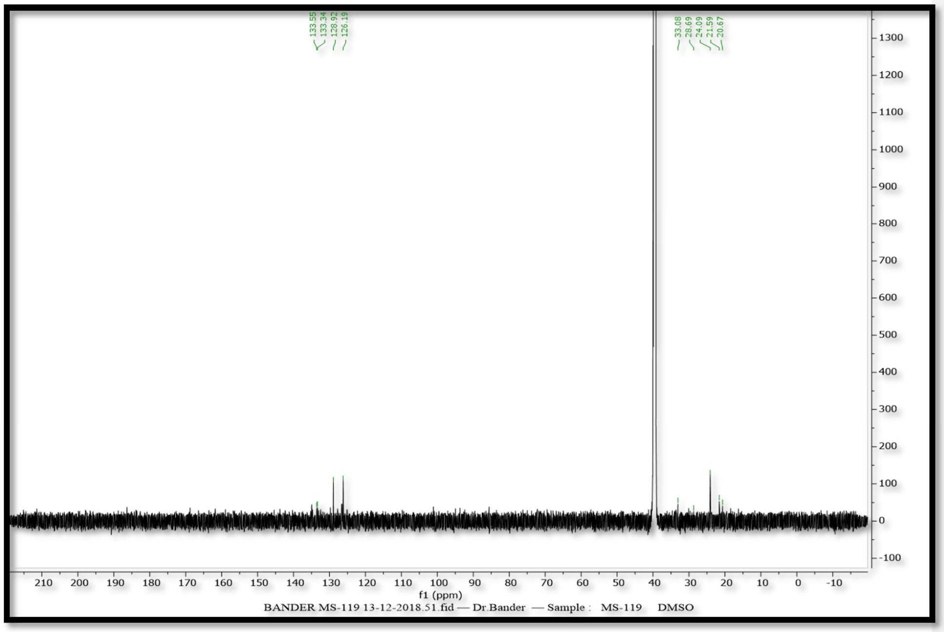

Figure S36: ^{13}C-NMR spectrum of [(η^6-*p*-cymene)Ru(1,2-diaminoanthraquinone)]PF_6

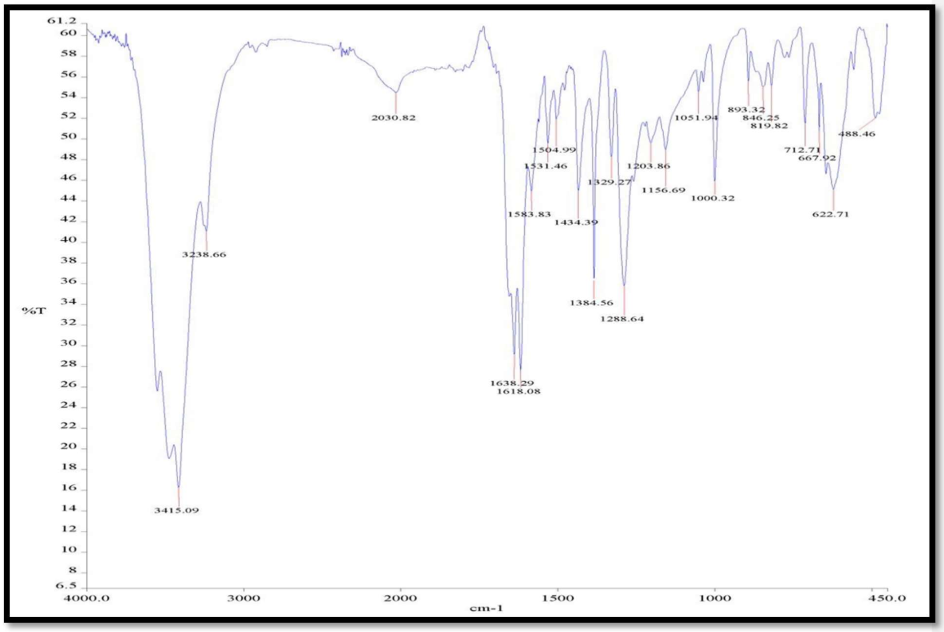

Figure S37: IR spectrum of [(η^6-*p*-cymene)Ru(1,2-diaminoanthraquinone)]PF_6

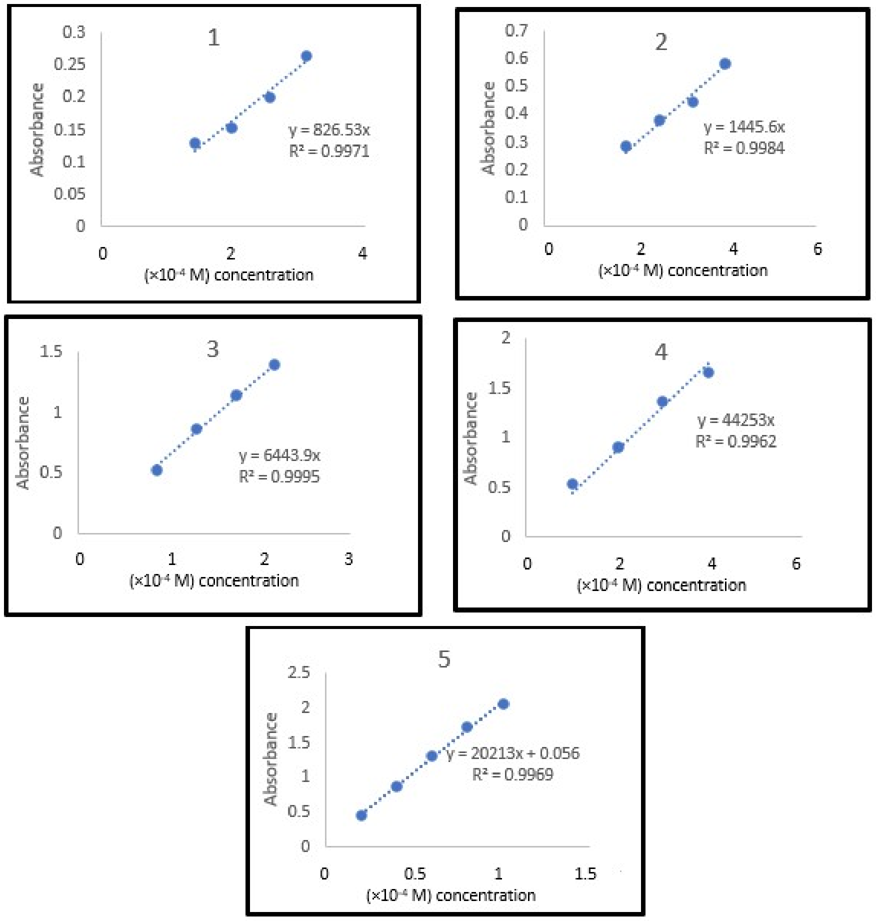

Figure S38: The determination of extinction coefficients of 1 -5.

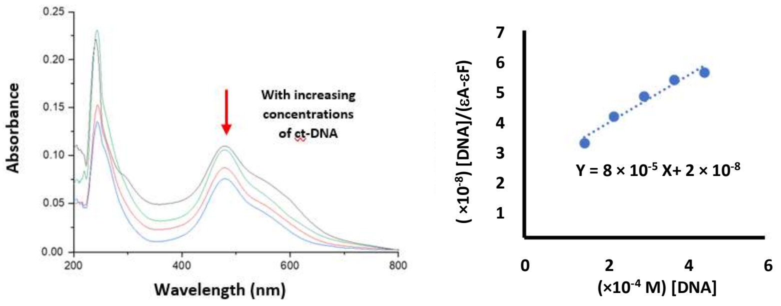

Figure S39: The titration of complex **1** with ct-DNA to determine the binding affinity at 490 nm.

Commented [M1]: According to our layout format, aEb should be written as a × 10b. Please revise.

Commented [BAMB2R1]: done

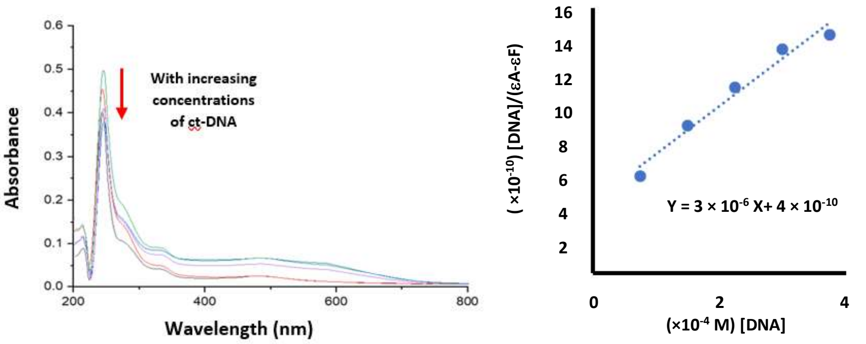

Figure S40: The titration of complex **2** with ct-DNA to determine the binding affinity at 350 nm.

Commented [M3]: According to our layout format, aEb should be written as a × 10b. Please revise.

Commented [BAMB4R3]: done

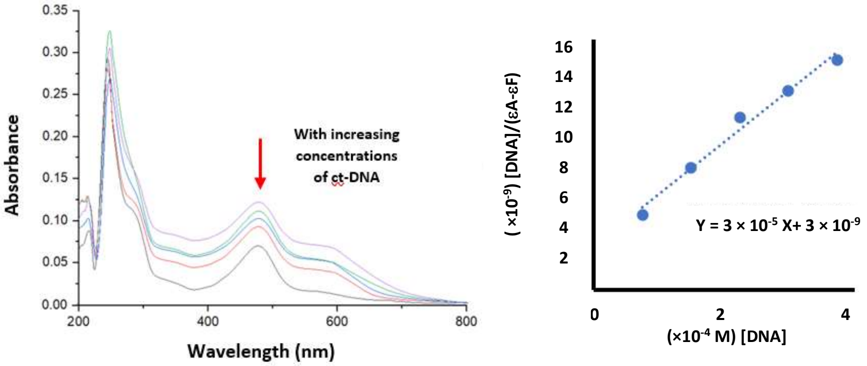

Figure S41: The titration of complex **3** with ct-DNA to determine the binding affinity at 480 nm.

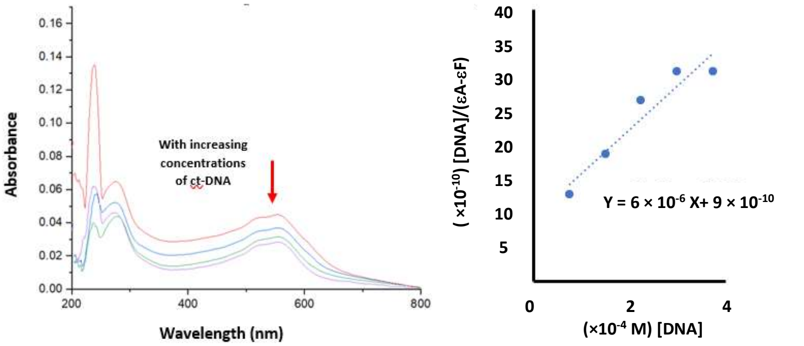

Figure S42: The titration of complex **4** with ct-DNA to determine the binding affinity at 565 nm.

Commented [M5]: According to our layout format, aEb should be written as a × 10b. Please revise.

Commented [BAMB6R5]: done

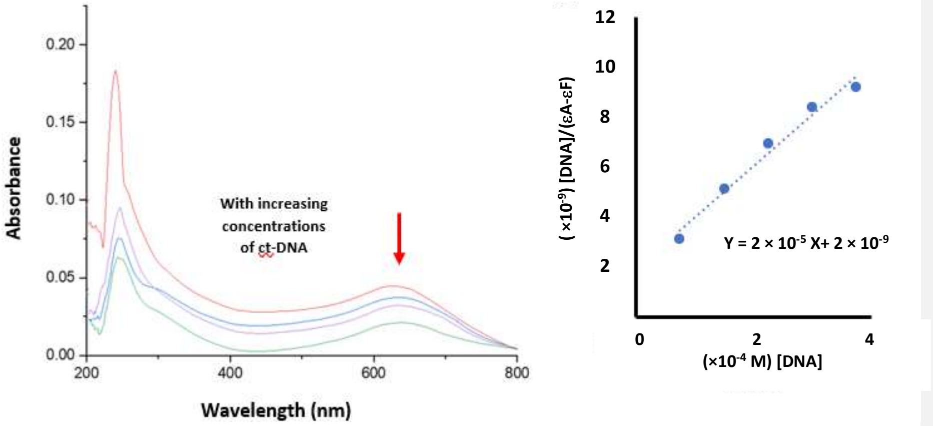

Figure S43: The titration of complex **5** with ct-DNA to determine the binding affinity at 640 nm.

Commented [M7]: According to our layout format, aEb should be written as a × 10b. Please revise.

Commented [BAMB8R7]:

Commented [BAMB9R7]: done

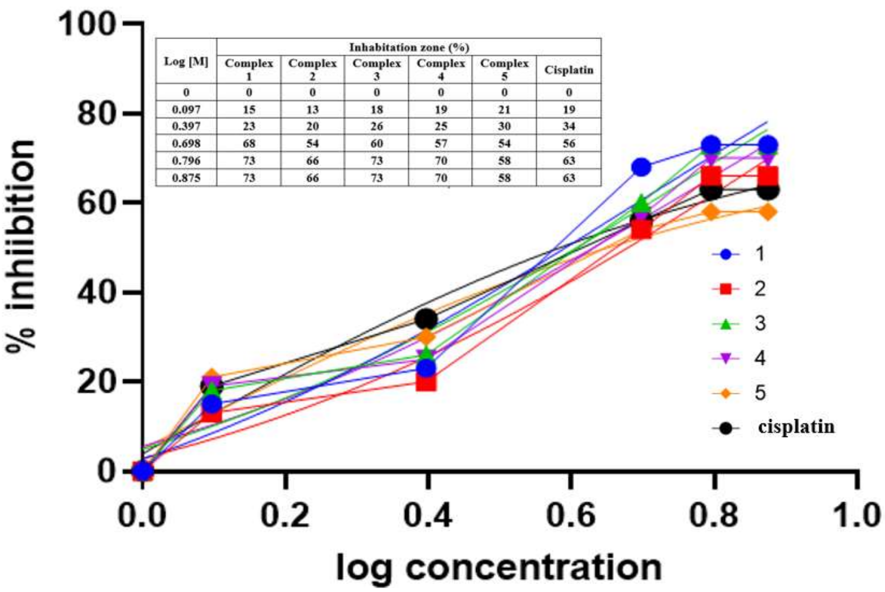

Log [M]	Inhabitation zone (%)					
	Complex 1	Complex 2	Complex 3	Complex 4	Complex 5	Cisplatin
0	0	0	0	0	0	0
0.097	15	13	18	19	21	19
0.397	23	20	26	25	30	34
0.698	68	54	60	57	54	56
0.796	73	66	73	70	58	63
0.875	73	66	73	70	58	63

Figure S44: The cytotoxicity of complexes **1–5** against OVCAR-3 cell line.

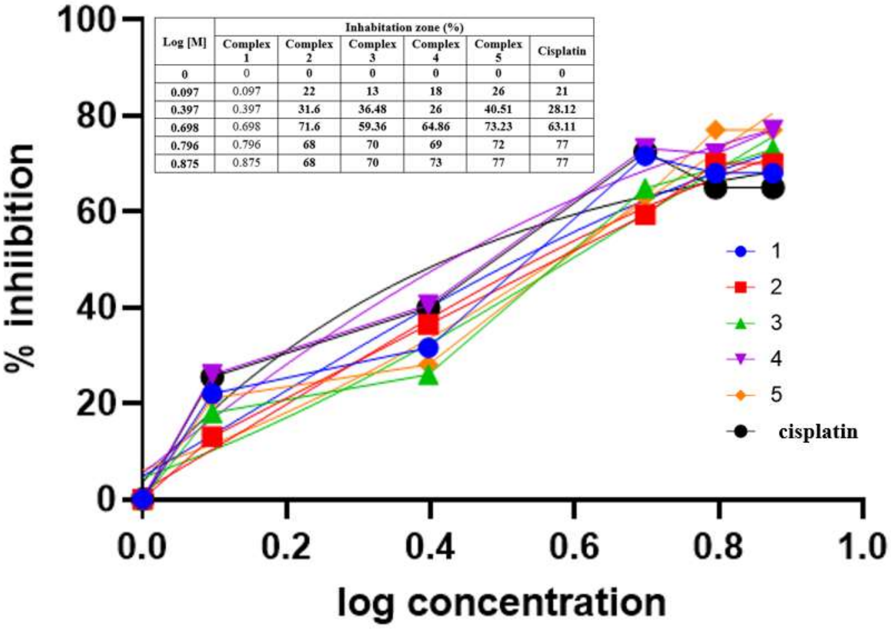

Figure S45: The cytotoxicity of complexes **1–5** against M-14 cell line.

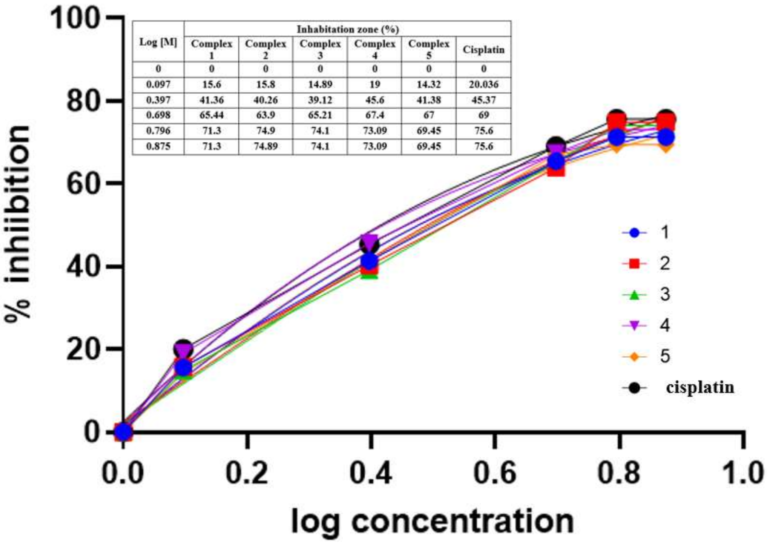

Figure S46: The cytotoxicity of complexes **1–5** against HOP-62 cell line.

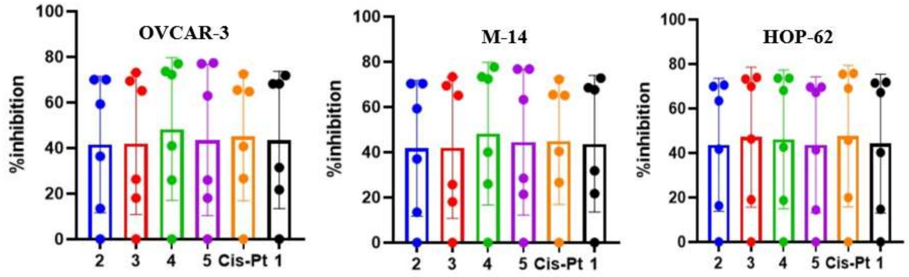

Figure S47: Inhabitation zones of the tested complexes at 1.25 µM, 2.5 µM, 5 µM, 6.25 µM and 7.5 µM of the compounds.

Cartesian coordinates of the complexes:

Structure1

Ru	-0.797092	-0.520310	-0.311781
C	-1.540241	2.309225	-0.274456
C	-1.503567	1.952667	1.079397
C	-2.328278	2.585604	1.997631
C	-3.212967	3.576613	1.548732
C	-3.249319	3.931382	0.201277
C	-2.401666	3.300964	-0.720728
H	-2.292563	2.310358	3.048498
H	-3.870261	4.068277	2.258725
H	-3.934677	4.700237	-0.140794
H	-2.420727	3.579498	-1.771155
N	-0.589382	0.869348	1.388946
H	0.366006	1.259495	1.371348
N	-0.660892	1.527408	-1.124055
H	0.300660	1.868043	-0.972375
H	-0.900362	1.619427	-2.109725
H	-0.770054	0.461319	2.304251
C	-1.016285	-2.701350	-0.820414
C	-1.498357	-1.916848	-1.900970
C	-2.512333	-0.933921	-1.682813
C	-3.037432	-0.773950	-0.369148
C	-2.478479	-1.511834	0.703358
C	-1.475885	-2.520273	0.510662
H	-0.160944	-3.336970	-1.005857
H	-1.059012	-2.038514	-2.885235
H	-3.775163	-0.003663	-0.170033
H	-2.817964	-1.292596	1.712565
C	-3.013044	-0.076747	-2.814118
H	-2.247270	0.052613	-3.585736

H	-3.876493	-0.560380	-3.286701
H	-3.338269	0.905861	-2.457407
C	-0.951174	-3.323012	1.698645
H	-1.836709	-3.778538	2.166555
C	-0.282770	-2.413614	2.748359
H	-0.970534	-1.646291	3.125978
H	0.041974	-3.005037	3.610325
H	0.593493	-1.919901	2.313863
C	0.003774	-4.451708	1.287042
H	0.297011	-5.026722	2.170883
H	-0.469084	-5.144284	0.581929
H	0.909225	-4.046362	0.822193
N	6.131447	1.836519	0.033992
C	4.915573	1.218349	-0.028426
N	4.836320	-0.049505	-0.366053
C	3.573078	-0.514398	-0.457051
N	3.189171	-1.777479	-0.798629
C	1.810538	-1.934950	-0.793248
N	1.309842	-0.720466	-0.429507
C	2.378216	0.172842	-0.216976
C	2.471915	1.534096	0.144208
O	1.546468	2.349071	0.386955
N	3.800433	1.988279	0.220068
H	6.923493	1.209964	-0.028916
H	6.258777	2.632190	0.644347
H	3.890217	2.978286	0.423312
H	3.833181	-2.518779	-1.037218

Structure2

Ru	-0.261804	-1.014397	-0.369765
C	6.643171	1.686242	-0.114337
C	5.528147	1.615084	-0.922597
C	4.254466	1.325939	-0.371380
C	4.141136	1.105944	1.041503
C	5.306485	1.185402	1.844946
C	6.531472	1.470162	1.279255
C	3.084504	1.242151	-1.183039
C	2.861816	0.808088	1.598385
C	1.763533	0.726370	0.786958
C	1.876153	0.945400	-0.614020
H	3.165537	1.410549	-2.254541
H	7.613449	1.911004	-0.546854
H	5.612827	1.784098	-1.993041
H	5.220123	1.020912	2.915898
H	7.417082	1.530857	1.904558
H	2.772951	0.640214	2.669343
N	0.636615	0.749179	-1.339435

H	0.792300	0.669284	-2.342872
H	0.028703	1.562042	-1.149587
N	0.429957	0.350709	1.215349
H	0.421782	-0.037625	2.156556
H	-0.159917	1.196814	1.189779
C	0.730433	-2.697636	0.641594
C	1.495518	-2.417684	-0.517482
C	0.850872	-2.340421	-1.784971
C	-0.542180	-2.645635	-1.865524
C	-1.266098	-3.003245	-0.697291
C	-0.664118	-3.032710	0.587964
H	1.221946	-2.640972	1.609666
H	2.540525	-2.141304	-0.423072
H	-1.065119	-2.563316	-2.812286
H	-2.340154	-3.098205	-0.782891
C	1.625345	-1.934441	-3.009980
H	2.073697	-2.822462	-3.471633
H	0.974401	-1.468967	-3.756995
H	2.438433	-1.246184	-2.757439
C	-1.419803	-3.388139	1.865505
H	-0.855855	-4.213014	2.326117
C	-2.850265	-3.877785	1.603349
H	-2.864569	-4.743607	0.932078
H	-3.314074	-4.181401	2.547124
H	-3.459114	-3.082578	1.159445
C	-1.430560	-2.213642	2.863269
H	-1.933183	-2.507073	3.790371
H	-0.415475	-1.895899	3.133594
H	-1.964875	-1.360721	2.430872
N	-4.863111	4.652010	0.282372
C	-4.155677	3.501487	0.078813
N	-4.785945	2.370092	-0.144666
C	-3.970391	1.300799	-0.254267
N	-4.342323	0.008722	-0.479760
C	-3.256851	-0.853625	-0.549433
N	-2.159194	-0.068139	-0.360650
C	-2.573586	1.265442	-0.176803
C	-1.904322	2.486485	0.053297
O	-0.670169	2.699027	0.164477
N	-2.783497	3.576823	0.174445
H	-5.850889	4.576642	0.076013
H	-4.443853	5.543686	0.056339
H	-2.327831	4.455257	0.398435
H	-5.300835	-0.292731	-0.586995

Structure3

Ru	-1.186697	-1.355100	0.137042

C	4.556221	-1.857523	-2.639572
C	3.294883	-1.809320	-2.076816
C	3.062987	-1.098727	-0.876337
C	4.137914	-0.404852	-0.242120
C	5.414249	-0.480070	-0.845324
C	5.623531	-1.190820	-2.013711
C	1.761547	-1.056625	-0.266070
C	3.886441	0.359094	0.974541
C	2.569091	0.400348	1.523710
C	1.529117	-0.349924	0.872914
C	2.311440	1.167278	2.684303
H	1.303508	1.229508	3.081786
C	3.327080	1.866935	3.307952
C	4.630799	1.818880	2.784655
C	4.898902	1.085040	1.642846
H	4.719789	-2.404950	-3.562801
H	2.473699	-2.314419	-2.576292
H	6.257189	0.029293	-0.393542
H	6.616844	-1.227267	-2.450674
H	3.116651	2.453793	4.196956
H	5.432119	2.364771	3.273518
H	5.912909	1.076926	1.261010
N	0.186022	-0.388234	1.431518
H	0.210376	-0.829518	2.352999
H	-0.203651	0.567047	1.578496
N	0.636697	-1.753943	-0.870638
H	0.850985	-2.744594	-0.998806
H	0.410328	-1.336894	-1.809040
C	-2.809689	-1.690122	1.646973
C	-1.981135	-2.861965	1.595070
C	-1.747362	-3.531140	0.376753
C	-2.356052	-2.988999	-0.810338
C	-3.173729	-1.845572	-0.746799
C	-3.457280	-1.177988	0.498488
H	-2.920057	-1.181238	2.596837
H	-1.493553	-3.211842	2.500559
H	-2.141746	-3.435247	-1.776944
H	-3.546045	-1.433318	-1.676284
C	-0.873104	-4.754449	0.300183
H	-1.492518	-5.657842	0.356996
H	-0.327564	-4.800195	-0.648736
H	-0.151974	-4.787271	1.122023
C	-4.459435	-0.028627	0.570404
H	-5.437348	-0.534560	0.623804
C	-4.479758	0.858603	-0.687043
H	-4.720209	0.300668	-1.596835
H	-5.249931	1.627838	-0.572969

H	-3.517432	1.355040	-0.834984
C	-4.316139	0.831098	1.837089
H	-5.056185	1.637059	1.814588
H	-4.502898	0.257586	2.751351
H	-3.320214	1.281559	1.902402
N	-1.034334	5.806961	-1.111611
C	-1.044653	4.440951	-1.069516
N	-0.912128	3.748180	-2.178789
C	-0.885528	2.411871	-1.984471
N	-0.705455	1.440956	-2.931085
C	-0.729413	0.164858	-2.378612
N	-0.951725	0.356993	-1.059350
C	-1.030054	1.721261	-0.778661
C	-1.093268	2.466168	0.424631
O	-1.088491	2.064993	1.607483
N	-1.136290	3.853499	0.175462
H	-1.091118	6.194744	-2.044573
H	-1.527660	6.329123	-0.400192
H	-1.114045	4.426143	1.012672
H	-0.552745	1.627653	-3.912314

Structure4

Ru	-1.100955	-1.030231	-0.389277
C	8.243972	0.662246	1.092810
C	7.034048	0.521806	1.721990
C	5.826124	0.598160	0.966998
C	5.900660	0.824581	-0.463143
C	7.180160	0.963732	-1.078212
C	8.317449	0.884493	-0.316673
C	3.618880	0.771629	-0.587902
C	3.544627	0.542818	0.837087
C	2.260759	0.395078	1.457926
H	2.227196	0.218226	2.529095
C	1.134247	0.469785	0.695591
C	1.208234	0.699119	-0.719771
C	2.406971	0.849257	-1.349821
H	9.164095	0.605496	1.666794
H	6.954210	0.353222	2.791014
H	7.212321	1.133486	-2.149552
H	9.291876	0.991990	-0.783873
H	2.484501	1.020350	-2.419637
N	4.791431	0.903636	-1.217982
N	4.645118	0.456070	1.592507
N	-0.212431	0.247941	1.174636
H	-0.707772	1.154633	1.161603
H	-0.227001	-0.134082	2.118150
N	-0.078620	0.657952	-1.379368

H	-0.589292	1.523342	-1.137101
H	0.016030	0.591256	-2.391467
C	-0.171952	-2.807964	0.522344
C	0.500840	-2.585771	-0.702269
C	-0.249851	-2.433905	-1.905807
C	-1.664153	-2.607891	-1.854748
C	-2.306123	-2.913772	-0.623058
C	-1.593231	-3.011462	0.598258
H	0.412717	-2.806696	1.438647
H	1.571215	-2.407866	-0.706302
H	-2.265622	-2.458760	-2.744807
H	-3.387558	-2.906514	-0.612076
C	0.443519	-2.083229	-3.194586
H	0.754015	-3.002787	-3.705252
H	-0.223149	-1.539577	-3.871463
H	1.343863	-1.486702	-3.014594
C	-2.260304	-3.306703	1.939089
H	-1.768997	-4.213435	2.323004
C	-3.762181	-3.595845	1.813777
H	-3.957320	-4.441232	1.144983
H	-4.171497	-3.849356	2.796625
H	-4.299720	-2.720646	1.431892
C	-2.017622	-2.178003	2.959985
H	-2.461461	-2.439427	3.925717
H	-0.948676	-2.003418	3.135463
H	-2.477668	-1.247647	2.607912
N	-5.100972	5.070789	0.289159
C	-4.522037	3.838149	0.194835
N	-5.266550	2.773039	-0.003619
C	-4.562907	1.631543	-0.148802
N	-5.064806	0.383677	-0.371551
C	-4.071391	-0.581117	-0.465449
N	-2.898377	0.090520	-0.291653
C	-3.175213	1.457174	-0.093469
C	-2.384661	2.604810	0.124746
O	-1.131692	2.695012	0.201861
N	-3.147259	3.776935	0.261368
H	-6.109969	5.055306	0.363267
H	-4.640917	5.803261	0.812159
H	-2.595804	4.622580	0.362919
H	-6.049988	0.178822	-0.464666

Structure5

Ru	-1.151409	-1.051626	-0.596239
C	7.199841	0.823830	1.932534
C	6.745308	0.968966	0.622623
C	5.380579	0.858813	0.338366

C	4.466503	0.595624	1.381134
C	4.933692	0.443281	2.693865
C	6.294330	0.560969	2.967660
C	4.922787	1.006387	-1.068179
C	3.017191	0.459517	1.116985
C	2.530529	0.713204	-0.271389
C	3.450519	0.919603	-1.329326
C	3.004777	1.054443	-2.643308
H	3.741665	1.213854	-3.422945
C	1.642356	0.969017	-2.944132
C	0.734781	0.764379	-1.914109
C	1.166245	0.671153	-0.582247
H	8.260169	0.913335	2.149003
H	7.429939	1.166686	-0.195361
H	4.217940	0.236941	3.482246
H	6.652147	0.447978	3.986563
H	1.297434	1.048398	-3.971580
O	2.235786	0.119104	2.014416
O	5.707887	1.197256	-1.987557
N	-0.682533	0.540980	-2.096046
H	-1.178073	1.395782	-1.791654
H	-0.915128	0.337128	-3.066274
N	0.105192	0.450454	0.367649
H	-0.436351	1.327321	0.429124
H	0.510308	0.214159	1.278776
C	-2.278552	-2.993246	-0.451140
C	-1.790044	-2.856767	-1.765492
C	-0.388744	-2.627016	-1.997708
C	0.482954	-2.572879	-0.882973
C	-0.044971	-2.635277	0.440644
C	-1.428846	-2.860827	0.693716
H	-3.349375	-3.035222	-0.290044
H	-2.480332	-2.881361	-2.602584
H	1.535989	-2.356577	-1.032635
H	0.628067	-2.450054	1.271872
C	0.134280	-2.455555	-3.398700
H	1.028615	-1.826080	-3.420594
H	0.401189	-3.434953	-3.813976
H	-0.625438	-2.020171	-4.056302
C	-2.022998	-2.955115	2.084703
H	-3.039385	-2.551403	1.996399
C	-1.272906	-2.119144	3.129918
H	-1.190872	-1.072108	2.816589
H	-1.815399	-2.143980	4.080120
H	-0.264895	-2.504671	3.325340
C	-2.113714	-4.435516	2.505359
H	-2.608565	-4.522489	3.478390

H	-2.687027	-5.024212	1.780649
H	-1.115132	-4.881225	2.592074
N	-5.116006	4.924175	0.914511
C	-4.519577	3.712205	0.711912
N	-5.198142	2.602518	0.899558
C	-4.509139	1.481594	0.601561
N	-4.961358	0.198186	0.680833
C	-3.998062	-0.729269	0.307967
N	-2.896541	0.003939	-0.016166
C	-3.186977	1.371170	0.155592
C	-2.471571	2.568140	-0.057132
O	-1.296977	2.719001	-0.483472
N	-3.219474	3.715627	0.255965
H	-6.020180	4.873862	1.366140
H	-4.551151	5.721997	1.172250
H	-2.743671	4.589620	0.058117
H	-5.895456	-0.055141	0.971390
